# Impact of Treatment with Antioxidants as an Adjuvant to Standard Therapy in Patients with Septic Shock: Analysis of the Correlation between Cytokine Storm and Oxidative Stress and Therapeutic Effects

**DOI:** 10.3390/ijms242316610

**Published:** 2023-11-22

**Authors:** Israel Pérez-Torres, Alfredo Aisa-Álvarez, Sergio Casarez-Alvarado, Gabriela Borrayo, Ricardo Márquez-Velasco, Verónica Guarner-Lans, Linaloe Manzano-Pech, Randall Cruz-Soto, Omar Gonzalez-Marcos, Giovanny Fuentevilla-Álvarez, Ricardo Gamboa, Huitizilihuitl Saucedo-Orozco, Juvenal Franco-Granillo, María Elena Soto

**Affiliations:** 1 Cardiovascular Biomedicine Department, Instituto Nacional de Cardiología Ignacio Chávez, Juan Badiano No. 1, Col. Sección XVI, Mexico City 14380, Mexico; pertorisr@yahoo.com.mx (I.P.-T.); loe_mana@hotmail.com (L.M.-P.); 2 Critical Care Department, American British Cowdray (ABC) Medical Center, PAI ABC Sur 136 No. 116, Col. las Américas, Mexico City 01120, Mexico; alfredoaisaa@gmail.com (A.A.-Á.); ogmarcos35@gmail.com (O.G.-M.); jfranco@abchospital.com (J.F.-G.); 3 Immunology Department, Instituto Nacional de Cardiología Ignacio Chávez, Juan Badiano No. 1, Col. Sección XVI, Mexico City 14380, Mexico; secazalv@gmail.com (S.C.-A.); marquezric@hotmail.com (R.M.-V.); randallcruz44@hotmail.com (R.C.-S.); 4 Instituto Mexicano del Seguro Social, Dirección de Prestaciones Médicas Coordinación de Innovación en Salud, Ciudad de México 06700, Mexico; gabriela.borrayo@imss.gob.mx; 5 Physiology Department, Instituto Nacional de Cardiología Ignacio Chávez, Juan Badiano No. 1, Col. Sección XVI, Mexico City 14380, Mexico; gualanv@yahoo.com (V.G.-L.); fuentevilla_alvarez@hotmail.com (G.F.-Á.); rgamboaa_2000@yahoo.com (R.G.); 6 Cardioneumology Department, Specialty Hospital, National Medical Center “La Raza”, Mexico City 02990, Mexico; huitzilihuitls@outlook.com; 7 Research Direction Instituto Nacional de Cardiología Ignacio Chávez, Juan Badiano No. 1, Col. Sección XVI, Mexico City 14380, Mexico; 8 Cardiovascular Line in American British Cowdray (ABC) Medical Center, PAI ABC Sur 136 No. 116, Col. Las Américas, Mexico City 01120, Mexico

**Keywords:** antioxidants, septic shock, melatonin, cytokines

## Abstract

Cellular homeostasis is lost or becomes dysfunctional during septic shock due to the activation of the inflammatory response and the deregulation of oxidative stress. Antioxidant therapy administered alongside standard treatment could restore this lost homeostasis. We included 131 patients with septic shock who were treated with standard treatment and vitamin C (Vit C), vitamin E (Vit E), N-acetylcysteine (NAC), or melatonin (MT), in a randomized trial. Organ damage quantified by Sequential Organ Failure Assessment (SOFA) score, and we determined levels of Interleukins (IL) IL1β, Tumor necrosis factor alpha (TNFα), IL-6, monocyte chemoattractant protein-1 (MCP-1), Transforming growth factor B (TGFβ), IL-4, IL-10, IL-12, and Interferon-γ (IFNγ). The SOFA score decreased in patients treated with Vit C, NAC, and MT. Patients treated with MT had statistically significantly reduced of IL-6, IL-8, MCP-1, and IL-10 levels. Lipid peroxidation, Nitrates and nitrites (NO_3_^−^ and NO_2_^−^), glutathione reductase, and superoxide dismutase decreased after treatment with Vit C, Vit E, NAC, and MT. The levels of thiols recovered with the use of Vit E, and all patients treated with antioxidants maintained their selenium levels, in contrast with controls (*p* = 0.04). The findings regarding oxidative stress markers and cytokines after treatment with antioxidants allow us to consider to future the combined use of antioxidants in a randomized clinical trial with a larger sample to demonstrate the reproducibility of these beneficial effects.

## 1. Introduction

Sepsis and septic shock are global health problems and their prevalence generates a high economic cost; 51% of patients are admitted to intensive care units (ICUs), and 17.3% are treated in intermediate care (IC) or coronary units (UCCs) [1]. Approximately 80% of patients have a risk of developing multiple organ failure (MOF) [2].

In patients with sepsis and septic shock, dysfunction and loss of cellular and metabolic homeostasis are conditioned by the patient’s response to the infection and are associated with a high mortality [3,4,5]. Since viruses, bacteria, and fungi cause infections leading to these conditions, the immune response must be rapid and coordinated; however, when the immune response is dysregulated or excessively triggered, a state of hyperinflammation occurs [6,7,8]. Several damage mechanisms interact, and it is vital to control the inflammatory response and the pro-oxidant state during treatment [9], because their deregulation leads to endothelial dysfunction, capillary permeability alterations, and variations in the coagulation system [10].

Immunoregulation by cytokines is vital; interferons mediate natural immunity, activate mononuclear phagocytes and neutrophils, and increase the molecules of the major histocompatibility complex. During septic shock, the participation of cytokines, such as IL-2 and IL-4, regulate the activation, proliferation, and differentiation of leukocytes [11,12,13]. Chemokines promote the adhesion of monocytes and neutrophils [14], and interferon-gamma (IFN γ) acts as a potent activator of mononuclear phagocytes and neutrophils to increase the expression of MHC class I and class II molecules [15]. TNF-α activates mononuclear neutrophils, and eosinophils, it acts on hepatocytes to induce the synthesis of acute-phase proteins and constitutes a central mediator of responses of the host against Gram-negative bacteria [16].

Similarly, IL-6 mediates natural immunity since it induces the synthesis of acute-phase proteins in the liver and is a proliferation factor for activated B cells. Its levels increase in patients with infection and are associated with mortality in patients with sepsis [17,18,19]. Likewise, elevated IL-6, IL-8, and TNF-α concentration are associated with septic shock and multiple organ failure [20,21,22,23,24,25]. The measurement of IL-8 identifies serious infections in patients with neutropenia, disseminated intravascular coagulation (DIC), lactic acidosis, severe hypoxemia, and high risk of mortality [26,27].

The endothelium actively participates in the pathophysiology and damage associated with sepsis, leading to systemic inflammation [28]. Interleukin 1 (IL-1) acts on the vascular endothelium and mononuclear phagocytes, activating endothelial functions, and it can lead to loss of anticoagulant properties and vaso permeability [20]. In perfusion models, elevated levels of Il-2 are associated with capillary leak syndrome where it activates neutrophils adhered to endothelial cells leading to the production of oxygen radicals [20]. Similarly, intercellular adhesion molecule 1 (ICAM-1) promotes diapedesis and participates in endothelial damage. High levels are present in patients with septic shock [28].

The deregulation of the host response and multiorgan failure due to infection is attributed to the interaction of various mechanisms, where oxidative stress (OS) participates. Mitochondrial dysfunction plays a vital role in the pathogenesis of septic shock [29]. Modulation of mitochondrial function could be a possible therapeutic strategy in the management of sepsis in the future because mitochondrial dysfunction leads to MOF in septic patients [30].

Some cytokines regulate inflammation, such as interleukin 10 (IL-10), which inhibits the activity and production of TNF-α, IL-1, IL-2, and chemokines [20,21]. A change in the ratio of IL-6 to IL-10 levels might be a marker to evaluate the systemic inflammatory response [31].

Immune cells express pattern recognition receptors (PRRs) that rapidly initiate host defense responses upon detecting tissue damage or microbial infection. Intracellular damage-associated molecular proteins (DAMPs) mediate immune recognition of damaged tissue. Toll-like receptors (TLRs), a subfamily of PRRs, have emerged as crucial receptors for DAMP recognition and initiation of the inflammatory response. During sepsis, immune response activation occurs due to the release of high levels of DAMPs originating from invading microorganisms and damaged host tissue, leading to overstimulation of immune cells. This unbalanced response is known as a cytokine storm and transforms its function from fighting excess infection into damage due to inflammation [32].

Furthermore, the stimuli produced by the infection lead to cell apoptosis through the intracellular or extracellular pathway (intrinsic or extrinsic pathway). Intrinsically, proapoptotic members of the Bcl family regulate the exit of cytochrome c from mitochondria, such as Bid and Bax. Other Bcl members (Bcl-XL and Bcl-2) are anti-apoptotic and are critical in signaling pathways leading to apoptosis or cell survival. Endoplasmic reticulum stress is another mechanism of apoptosis triggered by intracellular stimuli and mediated by caspase 12. In the extrinsic way, activation occurs due to binding a ligand, a polypeptide of the TNF family, with its receptor. This leads to activating initiator caspases 2, 8, and 10. Once activated, the caspases are cleaved and activate other effector caspases (3, 6, and 7), which enhance the cleavage of cytoskeletal and nuclear proteins, leading to apoptosis cells. The immune system’s responsiveness is impaired during sepsis, and increased apoptosis of immune cells may explain the development of immune dysfunction. Therefore, it is necessary to eliminate a more significant number of apoptotic cells. Despite critical efforts in septic shock research, no therapy significantly modifies its outcome, and new treatments have led to several experimental studies focusing on the apoptotic process [33].

Cytokines [34] and reactive oxygen species (ROS) [35] play a catastrophic role in sepsis. Cell respiration, protein folding, or by-products of metabolism mainly produce some reactive oxygen species (ROS); others are mainly generated by NADPH oxidase [36]. Also, leukocytes are attracted to the affected sites after infection and release cytokines and ROS [37].

Recently, antagonistic therapies anticytokine therapy targeting (tumor necrosis factor alpha [TNF-alpha], interleukin-1 [IL-1]) and anti-endotoxin strategies have been proposed as important therapeutic targets in sepsis. They use antibodies against endotoxins or endotoxin receptor/carrier molecules (anti-CD14 or anti-LPS binding proteins) [38]. These therapies are based on the presence of an exponential increase in many cytokines in inflammatory conditions where a cytokine storm is present. Recent studies demonstrate the effectiveness and usefulness of an early start of comprehensive therapeutic management in sepsis [39].

On the other hand, many experimental studies of in vitro models of sepsis using mouse hepatocytes AML12 have been published. These cells were treated with lipopolysaccharide (LPS) to induce sepsis by hepatocyte injury. In cells pretreated with melatonin, the effects on oxidative stress, inflammation, mitophagy, mitochondrial biogenesis, and adenosine triphosphate (ATP) levels were reduced, and the mitochondrial quality was improved. This finding demonstrated that the antioxidant melatonin had potential benefic therapeutic effects in sepsis induced in the liver by injury, promoting mitophagy and stimulating the biogenesis of mitochondrial activity. These findings justify research to explore the precise effects, the underlying mechanisms, and the effectiveness of melatonin in the clinical setting [40].

In animal models of sepsis, there were significant anti-inflammatory, antioxidant, and anti-apoptotic effects with the use of Sulforaphane, which inhibits the TLR4/NF-κB signaling pathway. There was a reduction in the cardiac damage caused by sepsis [41]. This finding supports the idea that the use of antioxidant therapy together with standard therapy in sepsis might be beneficial.

During septic shock, the cytokine storm is caused by the invasion of pathogens and a sustained high level of cytokines, which alter homeostasis and cause potentially fatal organ dysfunction [42,43].

Hyperinflammation is a prooxidant state with increased expression of reactive oxygen species (ROS). [Superoxide (O_2_^−^), Hydroxyl radical (OH), Hydroperoxyl radical (OOH), Peroxyl radical (ROO)], RNS [Nitric oxide (NO), Nitrogen dioxide (NO_2_) radical] are present in the circulating immune cells and in the affected organs, [44,45]. Elevated levels of intracellular superoxide are produced by NADPH oxidase, cyclooxygenase-2 (COX-2), and xanthine oxidase in the mitochondria during sepsis. SOD neutralizes superoxides under physiological conditions, but not during sepsis. In mouse models, the inhibition of NOX-2 prevents organ damage induced by sepsis. The inhibition of COX-2 also leads to a decrease in peroxynitrite in the experimental model of sepsis [46,47,48]. These findings suggest the importance of limiting excessive ROS generation with antioxidants rather than their effect on hyperinflammation to prevent sepsis. The clinical value of this strategy could be better if tested in the clinical environment.

Clinical trials to control only inflammation in septic shock have not had overwhelming success for many years. However, new immunomodulatory therapies and cytokine blockers have shown great success in severe cases of COVID-19 [49]. Standard management in intensive care units solves many fundamental conditions associated with septic shock, such as the cardiometabolic conditions and hemodynamic imbalances. Many studies in animal and experimental models use antioxidant therapy in septic shock; however, few studies demonstrate that the use of antioxidant therapy added to standard management in human patients with septic shock reduces organ dysfunction, improves clinical conditions, and reduces oxidative stress [50]. For this reason, our objective was to analyze whether adding antioxidant therapy in a randomized fashion to the standard treatment applied to patients with septic shock helps control cytokine levels and oxidant stress markers, and improves organ dysfunction.

## 2. Results

### 2.1. Population Studied

A total of 131 patients were included in this study, of which 61 (47%) were male and 70 (53%) female. The median age was 68 (58–78) years. Table 1 shows the demographic characteristics of the patients according to their assigned antioxidant treatment.

According to the different conditions that patients may present when entering an Intensive Care Unit, such as the reason for admission, place of access, diagnosis, and location of the initial infection, we compare these variables between the antioxidant treatment groups to evaluate if they were homogeneous. We show no differences, which reveals the homogeneity of these clinical conditions (Table 2).

### 2.2. SOFA Score Assessment

The SOFA score was assessed in patients during the five days of antioxidant treatment to identify the impact of the addition of antioxidant therapy on organ damage. Supplementary Material S1 shows the reduction in the score. Statistical analysis using repeated measures (which evaluates changes over time) showed that there was a statistically significant reduction in the score in patients treated with Vit C from 8 to 3.5, (*p* = 0.001) between day 0 and day 5. The reduction with NAC was from 7 to 4, (*p* = 0.003) and with melatonin from 8 to 2 (*p*= 0.001). The patients who received Vit E and the control group also showed a decrease in the score; however, the difference was not statistically significant, and the final SOFA score remained high [50].

**Figure 1:**
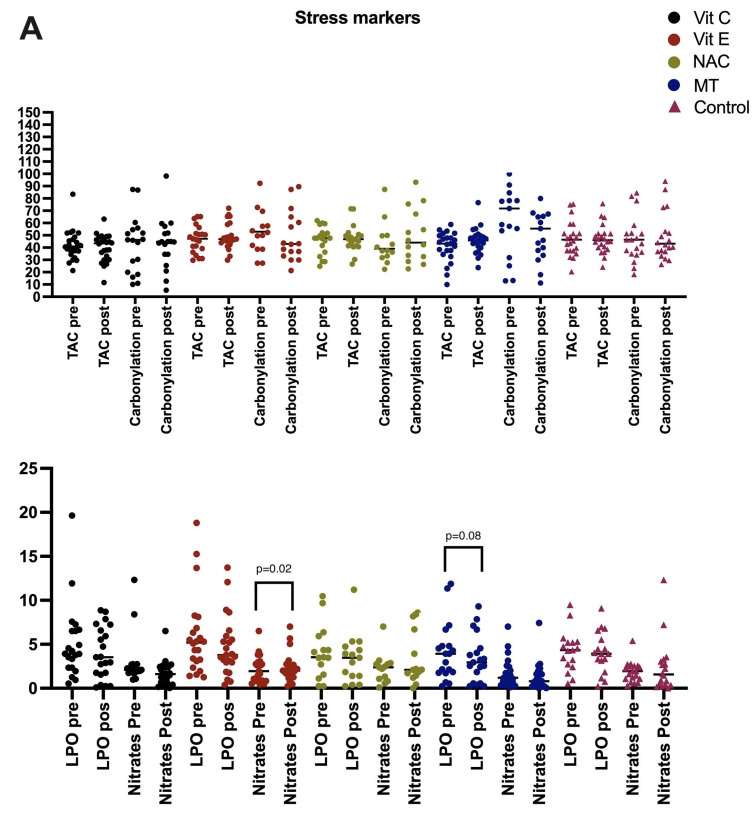

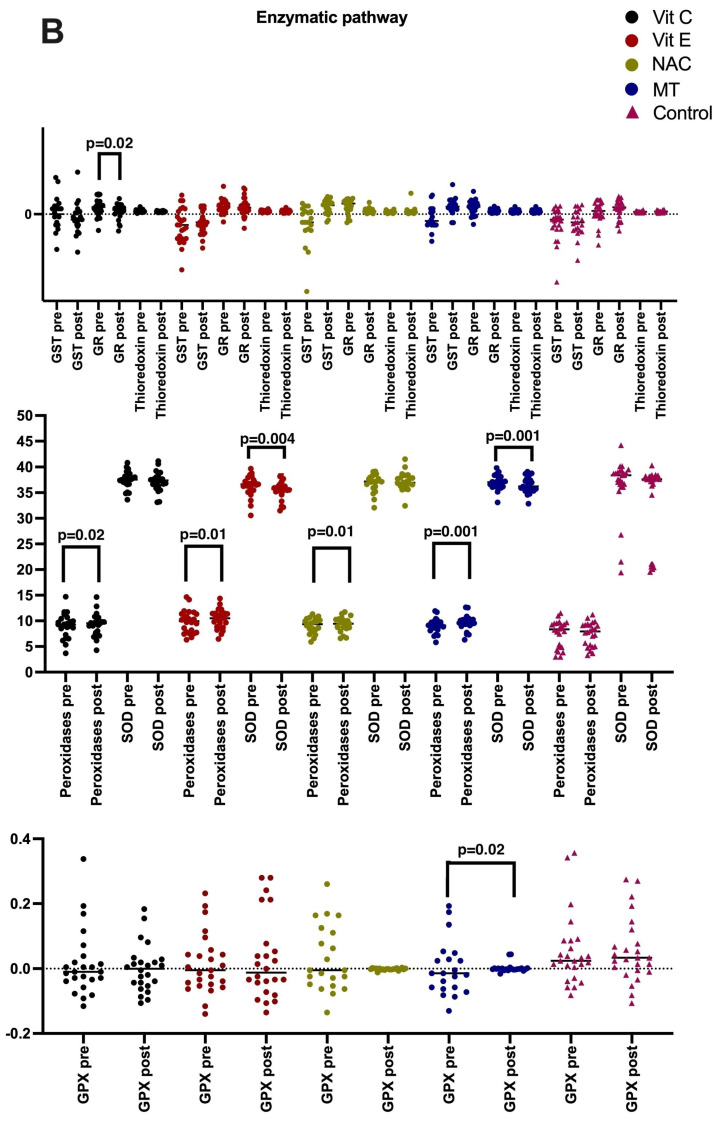

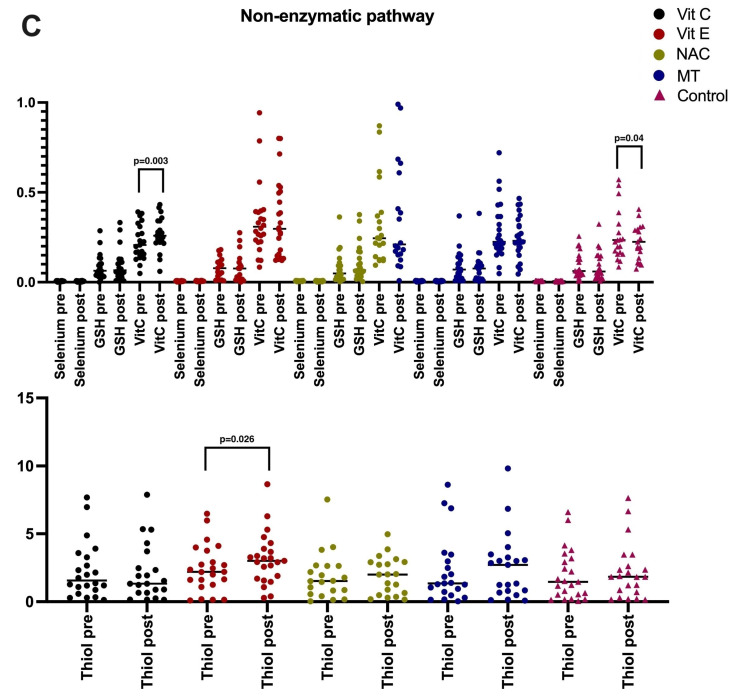
(**A**) Values of oxidative stress markers before and after antioxidant therapy. Groups: Vit C (*n* = 25), Vit E (*n* = 27), NAC (*n* = 24), MT (*n* = 26), and control (*n* = 29). Vit C: vitamin C; LPO: lipoperoxidation (nmol MDA/mL); TAC: total antioxidant capacity (TroloxnM/mL); Thiol (µM/mL); Nitrates and Nitrites (HNO_3_^−/^NO_2_^−^ nmol/mL of serum). Carbonylation (ngcarbonyl/mL); (**B**) Enzymatic pathway before and after antioxidant therapy. Peroxidases, (U/L) SOD: superoxide dismutase (U/mgL); (**C**) Enzymatic pathway before and after antioxidant therapy. GPX: glutathione peroxidase (µmol of NADPH/min/mL); GSH: glutathione (µM/mL of serum); GST: (µM/mg/protein); GR: glutathione reductase (U/min/mL Thioredoxin (µM/mg/protein).

In patients treated with antioxidants, selenium levels were maintained at the same level as at admission; however, in the control group, they decreased (Figure 1A–C).

### 2.3. Lipoperoxidation Levels: Enzymatic and Non-Enzymatic Antioxidant Pathways

Lipid peroxidation and oxidative stress markers were increased at the beginning of the treatment in patients with septic shock, and a decrease was observed in all patients who received antioxidant therapy. However, only MT showed a statistically significant change. NO_3_^−^ and NO_2_^−^ levels were elevated before treatment, and there was a statistically significant decrease in patients treated with Vit C. The antioxidant capacity of the patients showed low levels before treatment, and the levels increased with the use of Vit E, NAC, and MT; nevertheless, the difference was not statistically significant.

Regarding the enzymatic antioxidant pathway, we evaluated glutathione and found increased levels in patients treated with NAC *p* = 0.05. Glutathione peroxidase showed changes with the use of MT *p* = 0.02. SOD significantly decreased with the use of Vit E and MT *p* = 0.004 and *p* = 0.001, respectively. Glutathione reductase showed elevated levels before treatment and were decreased with the use of Vit C *p* = 0.02. Thioredoxins did not increase in the groups treated with antioxidants and the control group. All patients who received antioxidants had increased peroxidases, and the differences were statistically significant. The level decreased in the control group, but there was no statistical difference.

The levels of non-enzymatic antioxidants showed that there was an increase in thiols in patients treated with Vit E. All patients had low levels of Vit C but the only group that showed an increase was the one treated with Vit C *p* = 0.003.

### 2.4. Cytokine Quantification

**Figure 2:**
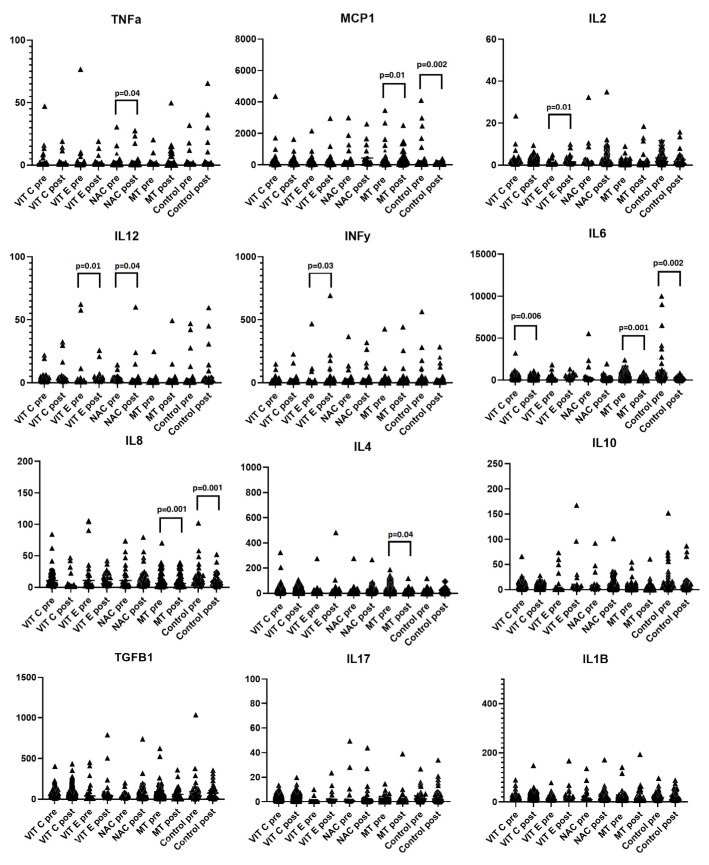
Proinflammatory interleukins show decreases with a significant delta when evaluating the pretreatment and posttreatment levels. With Vit C, there is a decrease in IL-6 (*p* = 0.006); with Vit E there is an increase in IL-2, IL-12, and IFNγ (*p* = 0.01, *p* = 0.01, and *p* = 0.03, respectively); with MT, there is an increase in MCP-1 and a decrease in IL-6, IL-8, and IL-4 (*p* = 0.001, *p* = 0.001, *p* = 0.01, and *p* = 0.04, respectively); and with NAC, there is a decrease in TNFα (*p*= 0.04) and an increase in IL-12 (*p* = 0.04). Individuals without antioxidant therapy had decreased levels of IL-6 (*p* = 0.002), IL-8 (*p* = 0.001), and MCP-1 (*p* = 0.002).

IL-1β, IL-2, IL4, IL-6, IL-8, IL-10, IL-12p70, IL-17A, TNF-α, INFγ, IP-10, MCP-1, and TGFβ-1 were quantified using the ELISA method. Table 3 shows the levels of proinflammatory and anti-inflammatory cytokines before and after treatment with the antioxidant therapy. Paired-sample *t*-tests showed significant differences between the pretreatment and posttreatment values. With the use of Vit C, IL-6 decreased (*p* = 0.006), while Vit E increased IL-2 and IL-12 (*p* = 0.01 for both), as well as IFNγ (*p* = 0.03). NAC decreased TNFα (*p* = 0.004) and increased IL-12 (*p* = 0.04), while MT increased MCP-1 (*p* = 0.01) and decreased IL-6, IL-8, and IL-4 (*p* = 0.001, *p* = 0.001, and *p* = 0.04, respectively). In the control group, there was an increase in MCP-1 (*p* = 0.002) and a decrease in IL-6 and IL-8 (*p* = 0.002 and *p* = 0.001, respectively). We also found that all antioxidants increased TGF-β; Vit C before treatment 44.7 (8.8–414.2) after 46.9 (0–532.2) *p* = 0.80, MT 45.09 (8.8–626.3) to 51.4 (8.8–359.6) *p* = 0.53, NAC 27.6 (8.8–411.6) to 52.9 (8.8–789.7) *p* = 0.08, control 45.1 (8.8–1033) to 75.8 (8.8–356-9) 0.67 except Vit E 63 (8.8–452.8) to 52.9 (0–826.7). The changes are shown in Figure 2.

### 2.5. Canonical Correlation

A canonical correlation analysis was conducted. It included clinical variables such as SOFA score, PCT PCR laboratory, and oxidant stress biomarkers, and considered explanatory variables (u1, u2, u3) and effect variables (v1, v2, v3).

The first evaluation of the canonical correlation carried out was with laboratory parameters and biomarkers. We included the explanatory variables and those of possible effect (cytocines). We found that in patients with septic shock, there is a high correlation of 0.95 when there are high levels of procalcitonin and carbonylation and low levels of total antioxidant capacity (TAC), glutathione, and vitamin C. This correlates with low levels of IL4 and elevated levels of IP10, IL1B, MCP1, IL-6, IL-17, and TNFα.

In the second evaluation of the canonical correlation, we found a correlation of 0.86 when there are high levels of LPO, carbonylation, and low levels of GSH, selenium, and thiols; this correlates with low levels of IL-4, il12p70, and IFNy and high levels of IL1B, mcp1, and IL-6. There was a high correlation of 0.82 (although lower than the two previous correlations) which shows us that high levels of the SOFA score, CRP, and CRP, and low levels of GSH and GSH peroxidase correlate with low levels of IL-4, TGFB1, and Il12p70 and high levels of IL1B and IL8 (Table 4).

### 2.6. Protein–Protein Interaction Network

**Figure 3:**
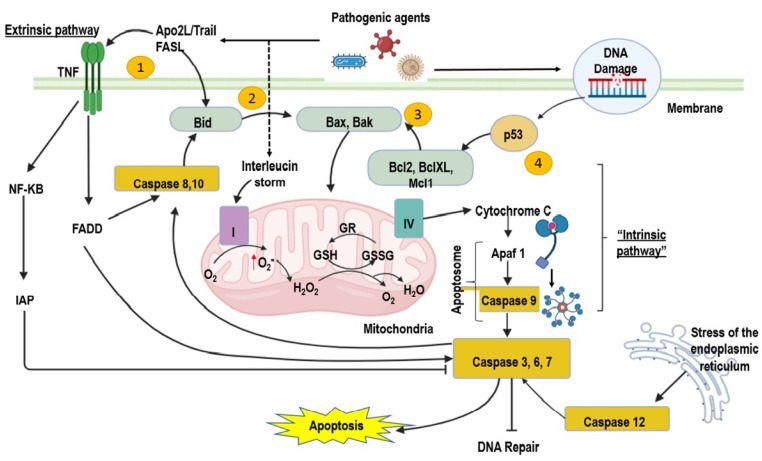
Intrinsic and extrinsic pathway for induction of apoptosis. Intrinsic and extrinsic pathway for induction of apoptosis. (1) The extrinsic pathway of apoptosis can be activated by TNF-dependent Apo2L/TRAIL receptors. (2) In some extional cases, Apo2L/TRAIL can be intrinsically pathway activated by Bid. (3) The intrinsic pathway can be activated by DNA or microtubule damage, involving the interaction of some proteins such as BAX and BAK. (4) Apoptosis can also be promoted by activation of p53. Abbreviations: Apaf-1 = apoptotic protease-activating factor 1, Apo2L/Trail = Recombinant human apoptosis ligand 2/inducing tumor necrosis factor-related apoptosis ligand 2, FADD = the fas-associated death domain protein, GR = glutathione reductase, GSH = glutathione, GSSG = oxidized glutathione, H_2_O_2_ = hydrogen peroxide, IAP = apoptosis inhibitor, NF-KB = kappa nuclear factor, O_2_^−^ = superoxide anion, TNF = tumor necrosis factor.

To present the participation of different mechanisms of damage in sepsis, we show the protein–protein interactions of the cytokines evaluated in this work; a diagram was created based on the Kyoto Encyclopedia of Genes and Genomes (KEGG), as well as the different signaling pathways that could be detected (Supplementary Material S2). The quantified interleukins might be involved in the presence of viral or bacterial infections that can lead to the development of septic shock. The interaction network was generated using the ShinyGO 0.76 server [51]. Also, we show in Figure 3 that in sepsis, the intrinsic and extrinsic pathways participate in the different mechanisms involved in damage to tissues and organs. And, we also explain our work on a hypothesis of inflammatory damage and oxidative stress through a graphical abstract.

## 3. Discussion

The control of the activation of pathophysiological mechanisms such as oxidative stress, and innate and adaptive inflammatory response in septic shock is vital [52], Deterioration and organ damage result from deregulation of physiological functions that lead to proteolysis [53,54] activation of the complement system, the coagulation pathway, the fibrinolytic system, lipid pathways, oxidative stress, and cytokine production [43].

On the other hand, the participation of pathogen-derived molecular patterns (PAMPs) and damage-associated molecular patterns (DAMPs), recognized by specific receptors on the cell surface, initiate the transcription cascade of inflammatory molecules due to the translocation of the nuclear factor NF-kβ in activated cells.

Imbalance requires early therapeutic management [55,56,57] since the cytokine storm leads to a potentially fatal result [42,57].

Cytokines activate and inhibit cellular functions by negatively regulating effector mechanisms and restoring homeostasis through the release of cytokines IL-10 and transforming growth factor-β. They also regulate protein deposition in the extracellular matrix and angiogenesis [58].

The effects of the use of antioxidant therapy in patients with septic shock and its effect on cytokines were among the objectives of this study since we have previously reported reduced biomarkers of oxidant stress with the use of this therapy. Furthermore, there was better disease control in patients with COVID-19. Although the results in previous works are good, experimental and clinical studies are still required to confirm this hypothesis [59].

In this sense, cytokines involved in innate immunity are attractive targets for therapeutic intervention [60]. They are essential in diseases whose pathogenesis is the result of a defective regulation of the cytokine network; therefore, cytokine-targeted therapies with monoclonal antibodies, soluble receptors, or small molecule inhibitors promise new possibilities for the treatment of patients resistant to standard drug regimens. The proposal to add antioxidants to standard therapy in septic shock is based on the reduction in oxidative stress and the increase in antioxidant capacity, and our group was one of the first to carry out studies in humans employing this therapy [50,60,61,62].

In this study, we support the hypothesis raised by us and other researchers that the function of cytokines changes with the use of antioxidant therapy. We found an increase in the level of IL-2 after treatment with Vit E, NAC, and MT. Only Vit E showed a statistically significant difference. This finding coincides with a previous one in children undergoing dialysis treatment, where they combined NAC with Vit E and demonstrated regulation of the cellular redox state and modulation of the cytokine profile. That study also found a reduction in organ damage at the level of the kidney in patients with septic shock [63]. IL-2 stimulates the proliferation of NK cells, increases their cytolytic function, and acts on B cells as a proliferation factor. Its increase is relevant since it acts as a stimulus for synthesizing antibodies and has a regulatory function.

The combined use of Vit E and NAC to stimulate the production of IL4 had a regulatory effect on oxidative stress, inflammation, and organ failure in experimental studies with an animal model [64]. In patients with septic shock, we found that, in addition to the attenuation of oxidative stress, there was an improvement in organic damage, and IL-4 levels increased in patients who received vitamin E. However, the delta did not reach statistical significance (*p* = 0.06).

NAC increased IL-10, which has regulatory properties. The increase in IL-10 levels due to the use of antioxidants is an important finding since there is multiorgan dysfunction that involves uncontrolled production of reactive oxygen species (ROS) and a cytokine storm in septic shock. At the same time, exhaustion of antioxidants that contribute to the progression to septic shock occurs. Macrophages secrete proinflammatory and anti-inflammatory mediators during the infectious process. The binding of lipopolysaccharide (LPS) to Toll-like receptor 4 (TLR4) releases TNF-α, which initiates proinflammatory events through tumor necrosis factor receptor 1 (TNFR1) signaling, which is an effect of IL-10 [65]. In humans with septic shock, the effect achieved with MT may be related to improving IL-10 levels.

IL-12 has a regulatory function and there was no association between its levels and severity of the disease in a study of children with septic shock [66]. In another study, IL12 levels were not modified in patients in a septic state after surgery [67]. The increase or decrease in IL-12 levels during septic shock could be measured longitudinally to determine its relevance in this condition. Unlike other studies, we found that Il-12 levels increased with Vit E and NAC therapy *p* = 0.001. This finding is relevant because this cytokine promotes cellular immunity by inducing the maturation of T cells into Th1 cells and stimulating the secretion of gamma interferon (IFN-γ), which activates natural killer cells, macrophages, and cytotoxic T cells. Therefore, its deficiency leads to deterioration of immune functions, favoring the spread of the infection.

The increase in IL-12 found in this study has particular importance given that the most important function of IL-12 is the induction of gamma interferon (IFN), which is a mediator in viral, fungal, bacterial, and parasitic resistance. The induction of IL-12 triggered by microbial products is a potent stimulus for IFNy, which leads to the quick control of the infection by these agents. However, if the response is not controlled, the synthesis of IL-12 can result in an excessive activation of the immune system which causes damage to the host tissue and even death. This occurs in autoimmune diseases and/or septic shock.

The Induction of IL-12 during acute infection is crucial to initiate the synthesis of INFy by NK cells and T lymphocytes, and mediators of resistance against viruses, bacteria, fungi, and parasites. In several animal models, IL-12 has been used as immunotherapy to protect cellular immunity and to control or reject inappropriate responses to pathogenic infections in organisms with immune disorders. In any case, increasing its concentration can be beneficial. However, the excess IL-12 response appears to be responsible for some problems observed during severe microbial infections. Under these conditions, IL-12 antagonists have attenuated immunopathology and prevented death. The toxic effects of the proposed therapy with IL-12 were observed in mice, and therefore its use as therapy in humans is controversial. A study with different doses is still necessary, or its combined use with antimicrobials with other molecules or with antioxidants [68].

In this study, IL-6 showed elevated levels before antioxidant therapy. Levels decreased in patients treated with Vit C, MT, and NAC. However, this effect was similar in the control group. This finding coincides with that from a systematic cohort study of patients with sepsis, in which the therapy was only with antibiotics and considered Il6 as a possible predictor of response to this therapy. The study also documented the substantial interindividual variability in the induction of IL-6 during sepsis since it depends on the type of infection, type of bacteria, and origin of the organ involved. All patients in our study received antibiotics. Therefore, the change and difference observed with antioxidants based on this cytokine will require an appropriate methodological strategy to discern whether the antioxidant effect creates synergy with the impact or is only related to the antibiotic [69].

Another regulatory cytokine involved in controlling the infectious process is TGF-β since it inhibits viral replication and cell proliferation. We found that all our patients had low levels of TGF-β before treatment, and levels increased in the group treated with antioxidants and in the control group. However, none showed a significant statistical difference; only the group treated with NAC had a clear trend of *p* = 0.08. Other studies have reported that its increase requires nutritional products such as sesame seeds and that it has synergistic properties with antioxidants such as vitamin E [70].

There is evidence for the complex pathogenesis of septic shock and LPS-induced re-actions. In recent years, mediators such as cytokines have aroused great interest for their role in inflammatory responses induced by LPS, TNFα, and IL-1 [71,72,73,74].

The relevance of TGFB1 in septic shock has been studied in mouse macrophages, and it inhibits JNK activity stimulated by lipopolysaccharide (LPS). It might have a possible inhibitory mechanism on TGF-beta in the signaling of inflammatory responses induced by LPS [75]. We found increased TGF-beta levels using all the antioxidants studied (Vit C, Vit E, NAC). In the control group, the levels decreased; it is precisely in this group of patients where SOFA remained elevated.

Several studies have demonstrated the involvement of proinflammatory cytokines in septic shock, and patients receiving standard treatment improve in intensive care units [76,77]; However, this response to treatment depends on the number of organs affected since admission [78] or if it was associated with surgical intervention, site of infection [79,80], burn [81,82], endocarditis [83,84], necrotizing fasciitis [85,86], meningitis [87,88], and septic arthritis [89,90,91]. The active participation of cytokines correlates with severity and prognosis [76].

High amounts of IL-1 and TNF-α are released during systemic inflammation, resulting in hypotension and shock. However, IL-1 is frequently undetectable and, when present, has little predictive value [92]. This study confirms this observation since the cytokines’ IL-1β and TNFα levels did not show relevant changes after treatment. In this regard, we consider that with our findings, we show that there are favorable changes in some cytokines with standard comprehensive management and antioxidant therapy. Comprehensive management probably requires exploring the synergistic therapeutic link of these pathways since there is information that with the use of some specific biologicals, the beneficial effect has not been fully proven in humans with infectious processes. During the COVID-19 pandemic, the use of anakinra, an IL-1 antagonist, or IL-6 antagonists were not wholly conclusive [93].

On the other hand, results using antioxidants at the experimental level have shown that some have regulatory capacity on oxidative stress. MT has radical scavenging properties and protects cell membrane lipids, cytosol proteins, nuclear proteins, and mitochondrial DNA. In our research, it reduced LPO, similar to Galley’s findings [94]. Also, the beneficial effect of MT has been demonstrated more widely in experimental cells, plants, and animals, although its mechanism of action remains unknown. The effects of MT could be related to its detoxifying capacity, thus protecting molecules from the destructive effects of OS in various conditions, such as ischemia/reperfusion (cerebrovascular accident and myocardial infarction), ionizing radiation, and drug toxicity. In sepsis, the protective effects of MT are associated with the inhibition of apoptosis and the reduction in OS [95]. MT has been shown to increase total antioxidant capacity (TAC) in experimental studies, equally with the associated use of Vit E and NAC [95,96,97]. On the other hand, the use of MT decreased the SOFA score, confirming the same effect found in multiorgan lesions induced by sepsis [98,99].

After release, plasma melatonin is rapidly distributed and transported to the mitochondria, acting as an antioxidant. In mammals, MT is an agonist of MT1 and MT2 receptors [100,101], and has pharmacological and physiological anti-inflammatory effects through receptor-dependent and non-receptor-dependent pathways [102]. It reduces levels of lipid peroxidation [103]. Also, we found an increase in GR activity in patients before treatment, which may lead to a decrease in GSH concentrations and an increase in ROS. GR activity decreased in patients treated with vitamin C, and GST activity remained unchanged in patients treated with Vit C and other antioxidants, but in the control they decreased. These findings on the activity of these enzymes with antioxidant treatment suggest that they could contribute to the reduced levels of LPO, and the SOFA score that evaluates MOF.

GPx uses selenoprotein P as a cofactor and decreases during septic shock [104]. We found low levels of Se in patients with septic shock and MOF. Although Se levels did not increase with the administration of antioxidant therapy, they were maintained, contrasting with the decreased levels found in untreated patients, who also showed no improvement in the SOFA score. The exact mechanism is not yet understood; however, our results suggest that the antioxidant therapy can maintain Se levels in septic shock patients, leading to improved GPx activity.

One of the inducers of sepsis is endotoxin or lipopolysaccharide (LPS), which comes from Gram-negative bacteria and leads to sepsis and multiorgan dysfunction [105]. LPS increases heart rate and decreases systemic vascular resistance, leading to hypotension. LPS also induces the synthesis of cytokines and elevates neutrophils, free radicals, and proteolytic enzymes that lead to endothelial dysfunction [106].

The glutathione peroxidase enzyme metabolizes intermediates formed by aerobic metabolism, such as hydrogen peroxide; this reaction occurs in the cytosol and mitochondria, resulting in oxidized glutathione, which is reduced by glutathione reductase. GR employs NADPH as a cofactor, helping to recycle an oxidation–reduction [107,108].

Glutathione is a tripeptide composed of three amino acids: glutamic acid, glycine, and cysteine. It plays a vital role in septic shock since it protects against damage caused by free radicals. Like glutathione, Vit E is a fat-soluble antioxidant [109]. It reacts with free radicals generated in the lipid phase and protects the lipids in the membranes. Vit C has radical scavenging activity, stabilizes membrane structures, oxidizes vitamin E, and has a synergistic mechanism [110].

In this study, Vit C and Vit E decreased LPO. Vit E and MT increased the total antioxidant capacity. NAC, a direct precursor of glutathione, increased glutathione levels and improved multiorgan failure (MOF), in accordance with previous research [111].

The effect of various antioxidants, such as NAC, MT, vitamins A, C, and E, enzyme cofactors (selenium and zinc), endogenous compounds (ubiquinone, α-lipoic acid, bilirubin, albumin, ferritin), and quercetin, can inhibit reactive nitrogen, oxygen species (ROS), and RNS [61]. The effect NAC has on improving antioxidant capacity is due to the replenishment of glutathione (GSH) and the sequestration of ROS. It also improves hemodynamic variables, cardiac index, oxygenation, and static lung compliance [62], and reduces the levels of IL-6 and ICAM-1 [60].

Innate cytokines are attractive targets for therapeutic intervention [63]. They are essential in diseases whose pathogenesis is the result of a defective regulation of the cytokine network; therefore, cytokine-targeted therapies with monoclonal antibodies, soluble receptors, or small molecule inhibitors promise new possibilities for the treatment of patients resistant to standard drug regimens [64,65]. The proposal to add antioxidants to standard therapy in septic shock has been previously proposed for its benefits in terms of reduction in oxidative stress and an increase in antioxidant capacity. Our group was one of the first to propose this [50,61].

In septic shock, interleukins are the first to increase, reaching a maximum peak at 2 h, when procalcitonin increases, and IL-6 and IL-8 levels decrease [112]. This aspect limits the interleukins’ determination during clinical practice; therefore, having little use in emergencies [112]. Moreover, as the deterioration of the organ progresses, determining cytokines, considering their lifespan, could add more information to clinical treatment. C-reactive protein (CRP) and procalcitonin (PCT) could be of help in the guidance of management. Determining oxidative stress markers is not routinely feasible today; however, this study and the experimental ones that show the participation of oxidative stress in the progression of damage in patients with septic shock allow us to suggest using antioxidant therapy associated with standard management.

In sepsis, organ dysfunction is potentially fatal, and it is caused by deregulation of the host response to infection. The pathological process is complex, and several cytokines participate in the damage mechanism, which, when dysfunctional, lead to an intertwined storm whose outcome worsens function and causes structural damage at the cellular, tissue, and organ level. The uncontrolled inflammation cascade requires regulation of both the cytokines involved and other intertwined mechanisms, such as the deregulation of oxidative stress, which requires simultaneous treatment and modulation to repair tissues and improve organ function.

Standard therapy in sepsis includes hydroelectrolytic hemodynamic management and antibiotic therapy that essentially control the initial inflammatory pathophysiological damage. However, deregulation of oxidative stress must also participate and is necessary to improve organ dysfunction and interact in the modulation of some cytokines.

Although there are limitations with using oxidative stress markers in emergency conditions, in this research we show that there is deregulation of oxidative stress in septic shock, which is related to cytokine storm, and that the complex interaction produced by treatment with antioxidants can improve organic dysfunction.

A limitation of this study is that the sample size would have to be larger to confirm the statistical power for all cytokines.

## 4. Methods and Materials

This study was a prospective, longitudinal, aleatorized, and blind clinical trial with a cohort of patients who were treated between April 2018 and January 2022. This dataset corresponds to the same population used in another study of our group and the study was completed with a new study of cytokines, which were not previously analyzed [50].

### 4.1. Study Population

Patients of any gender, over 18 years of age who were admitted to the intensive care unit of the ABC Observatory Medical Center and Santa Fe campus with a diagnosis of septic shock, according to the Third International Consensus on Sepsis and Septic Shock, were included. [73] The characterization of septic shock included refractory hypotension requiring the use of vasopressors despite resuscitation with sufficient fluids (20 mL/kg colloid or 40 mL/kg crystalloid) to maintain blood pressure ≥ 65 mmHg and lactate > 2 mmol/L. Patients were required to give a signed consent or an informed assent of the patient or responsible caregiver. Patients with a history of having signed an advance directive, chronic use of steroids and/or statins in the last six months, or with recent use of antioxidant treatment before the moment of septic shock, and who had any contraindication for the use of Vit C, Vit E, NAC, or MT, as well as pregnant women, were excluded and patients who, having already been included, withdrew their informed consent were removed from the study.

An advance directive is a legal instrument through which a competent person determines in writing their will regarding the treatment that they would and would not like to receive in a situation where they cannot express their will.

In standard therapy, patients receive various therapies; in general, they receive antibiotics, blood products, sedation and analgesia, steroids, and intravenous solutions. Hemodynamic support is monitored, and goals related to lactate control, glycemic control, cultures, mechanical ventilator withdrawal test, stress ulcer prophylaxis, and nutritional care are performed [72].

### 4.2. Sample Size

The sample size was calculated considering the mean difference between low levels of ascorbic acid and improvement in treatment with antioxidants. The test suggested the inclusion of 11 patients in each group for a desired power of 80% and an alpha error of <0.05 based on a previous study [74]. To achieve greater statistical power, the sample size was increased to >24 patients per group, and a power of 97% was reached with an alpha error of 0.03.

### 4.3. Randomization

Computerized electronic selection was used to divide the patients into blocks; Randomization was 1:1 in balanced blocks. There were 6 blocks, with an approximate number of 25 patients per block. Personnel who were not involved in the study participated in the blinding and placed the indicated therapy in identical opaque envelopes numbered from 1 to 125, and these were applied consecutively. Group 1 received Vit C, Group 2 received Vit E, Group 3 received NAC, Group 4 received MT, and Group 5 was the control group with antioxidant treatment. Of 3745 patients admitted to the ICU during this study, only 131 had septic shock. Once the diagnosis of septic shock was established (inclusion criterion), we applied treatment with antioxidants to patients according to randomization. We show the number of patients who entered each group and the type of treatment they received. Additionally, we provide the conditions that occurred during follow-up (intervention discontinuation, adverse effects, death) in each group and show the number of patients finally included in the analysis (Supplementary Material S3).

### 4.4. Masking and Drug Administration

The random assignment sequence for the administration of antioxidants was generated at the coordination center using a computer-generated randomization program. The pharmacy maintained the blinding. Blinding was also carried out from the study’s beginning until the results’ analysis. A pharmaceutical professional maintains surveillance and care of the form of application of each of the antioxidants according to the type of absorption and interactions with other medications and the most appropriate way to administer them under the conditions that each patient requires.

All antioxidants were administered orally or through a nasogastric tube for 5 days in addition to standard therapy. NAC was administered in 1200 mg tablets every 12 h. In addition, patients were given 50 mg of MT in 5 mg tablets once a day and vitamin C in 1 g effervescent tablets every 6 h. Vit E capsules of 400 IU were administered every 8 h. The preparation and administration of these drugs is presented in Supplementary Material S4. The doses of antioxidants were chosen according to what is reported in the literature [113,114,115,116].

The pharmacotherapeutic follow-up of the antioxidants used in the clinical trial had strict surveillance of the administration of medications by the CMABC Pharmacy Department to establish that antioxidants were administered as much as possible under the same conditions, both in the preparation and preparation administration hours (Supplementary Material S4).

### 4.5. Data Collection Method

Upon admission to the intensive care unit, a complete medical history and physical examination of the patients were performed to obtain the demographic data of the patients. APACHE II and SAPS II prognostic scores were calculated upon admission to the intensive care unit, and the SOFA score for organ dysfunction (neurological, respiratory, hemodynamic, hepatic, and hematological) was performed. Laboratory measurements were taken upon admission to the ICU and for every day of hospital stay, which included complete blood count, blood chemistry, serum electrolytes (sodium, potassium, chloride, calcium, and magnesium), liver function tests, C-reactive protein, procalcitonin, and venous and arterial blood gases.

The SOFA score was evaluated using 6 important systems, including respiration (PaO_2_/FiO_2_), coagulation (platelet count), liver (bilirubin), cardiovascular (mean arterial pressure), central nervous system (Glasgow Coma Scale, GCS), and renal system (creatinine and/or urine output) [117]. A detailed definition of the SOFA criteria including the relevant thresholds is shown in Supplementary Material S5.

### 4.6. Standard Therapy in the ICU

The patients were treated according to the recommendation of the International Guidelines for the Management of Sepsis and Septic Shock. This management includes fluid replacement, hemodynamic support, lactate goals, control of the infectious focus, antibiotic cultures, use of blood products, sedation and test for withdrawal from mechanical ventilation, use of steroids, glycemic control, prophylaxis of stress ulcers, and nutrition [77].

### 4.7. Sample Collection and Storage

Blood samples were obtained from each patient that entered the draw, before initiation of the treatment, and 48 h after its administration. The blood samples were centrifuged for 20 min at 936× *g* and 4 °C. The plasma of the samples was placed in 3 or 4 aliquots and stored at −30 °C.

### 4.8. Oxidative Stress Markers in Plasma

#### 4.8.1. NO_3_^−^/NO_2_^−^ Ratio

The NO_3_^−^ was reduced to NO_2_^−^ by the nitrate reductase enzyme reaction. A quantity of 100 µL of plasma previously deproteinized with 0.5 N, NaOH and 10%, ZnSO_4_ was mixed, and the supernatant was incubated for 30 min at 37 °C in presence of nitrate reductase (5 units). At the end of the incubation period, 200 µL of sulfanilamide 1% and 200 µL of N-naphthyl-ethyldiamine 0.1% were added and the total volume was adjusted to 1 mL. The absorbance was measured at 540 [118].

#### 4.8.2. GSH Concentration

A total of 100 µL of plasma previously deproteinized with 20% trichloroacetic acid (vol/vol) and centrifugated to 10,000× *g* for 5 min was added to 800 µL of phosphate buffer 50 mM, pH 7.3, plus 100 µL of 5, 50-dithiobis-2-nitrobenzoic acid 1 M. The mixture was incubated at room temperature for 5 min and absorbance was read at 412 nm [119].

#### 4.8.3. Evaluation Total Antioxidant Capacity (TAC)

Briefly, 100 µL of plasma were suspended in 1.5 mL of a reaction mixture prepared as follows: 300 mM acetate buffer pH 3.6, 20 mM hexahydrate of ferric chloride, and 10 mM of 2,4,6-Tris-2-pyridil-s-triazine dissolved in 40 mM HCl. These reactives were added in a relation of 10:1:1 *v*/*v*, respectively. After mixing, samples were incubated at 37 °C for 15 min in the dark. The absorbance was measured at 593 nm [120].

#### 4.8.4. Lipid Peroxidation (LPO)

A quantity of 50 µL CH_3_-OH with 4% butylated hydroxytoluene plus phosphate buffer pH 7.4 was added to 100 µL of plasma. It was incubated and centrifuged at 4000 rpm in room temperature for 2 min. Then, the n-butanol phase was extracted, and absorbance was measured at 532 nm [118].

#### 4.8.5. Carbonylation Protein Concentration

Briefly, 100 µL of plasma were added to 500 µL of HCl 2.5 N in parallel with another sample with 500 µL of 2, 4-dinitrophenylhydrazine (DNPH) and incubated. At the end of the incubation period, they were centrifuged at 15,000× *g* for 5 min. The supernatant was discarded. Two washings were performed. The mixture was incubated again at 37 °C for 30 min. Absorbance was read in a spectrophotometer at 370 nm, using bi-distilled water as blank and a molar absorption coefficient of 22,000 M^−1^ cm^−1^ [118].

#### 4.8.6. Determination of Selenium (Se)

Selenium (Se) determination was performed using 200 µL of serum according to the method described by Soto et al., and the absorbance was read at 600 nm [118].

#### 4.8.7. Thiols

This test consists of Ellman’s reagent reaction with a thiol group, commonly a thiolate, producing thiol-nitrobenzoate through potassium hydride. The technique used was previously described by Erel and Neselioglu [121], with some modifications carried out in our laboratory as previously reported [122]. A quantity of 50 µL of serum was used for the determination and the absorbance was measured at 415 nm. The calibration curve was obtained with solution GSSG 1 mg/1 mL and the absorbance was measured at 415 nm.

#### 4.8.8. GPx Activity

A total of 100 µL of serum was suspended in 1.6 mL of 50 mM phosphate buffer (KH_2_PO_4_, pH 7.3), 0.2 mM NADPH, 1 mM GSH, and 1 IU/mL glutathione reductase. The mixture was incubated for 3 min at 37 °C; then, 100 µL of 0.25 mM H_2_O_2_ was added to start the reaction and the absorbance was monitored for 7 min at 340 nm [123]. The units are expressed in µmol NADPH oxidized/min/mL in serum with an extinction coefficient of 6220 M^−1^ cm^−1^ at 340 nm of NADPH [124].

#### 4.8.9. GST Activity

A total of 100 µL of serum was added to 700 µL of phosphate buffer (KH_2_PO_4_, 0.1 M, pH 6.5) with 100 µL of 0.1 mM GSH and 100 µL of 0.1 mM 1-chloro-2,4-dinitrobenzene (CDNB). The sample was incubated and monitored for 7 min at 37 °C and read at 340 nm. GST activity was expressed in units of GS-DNB µmo/min/mL of serum with an extinction coefficient of 14,150 M^−1^ cm^−1^.

#### 4.8.10. Extracellular Superoxide Dismutase (ecSOD)

ecSOD activity was determined via electrophoresis using native 10% polyacrylamide gels. Electrophoresis was carried out at 120 V for 4 h, as previously described by Pérez-Torres et al. [39]. In brief, 100 L of serum were used; the gel was incubated in 2.45 mM NBT solution for 20 min. The liquid was discarded, and then the gel was incubated in a TEMED solution with 36 mM potassium phosphate (pH 7.8) and 0.028 mM riboflavin. The gel was exposed to a UV lamp for 10 min and washed with distilled water to stop the reaction. A standardized curve was obtained using a serial dilution (2.5, 5, 10, 15, 30, and 60 ng) with SOD from bovine erythrocytes (Sigma Aldrich Chemical SA de RL de C.V., Toluca, México). SOD activity was calculated.

#### 4.8.11. Vitamin C Levels

Briefly, 100 µL of 20% trichloroacetic acid were added to 100 µL of plasma and centrifuged at 5000 rpm for 5 min. Then, 200 µL of Folin–Ciocalteu reagent 0.20 mM was added to the supernatant. The mixture was incubated for 10 min. The absorbance was measured at 760 nm [103,125].

### 4.9. Cytokine Measurement

Serum cytokine levels were determined using a flow cytometry bead-based LEGENDplex™ HU Essential Immune Response Panel (BioLegend, San Diego, CA, USA), a 13-plex human panel to analyze the following cytokines and chemokines: IL-1β, IL-2, IL-4, IL-6, IL-8, IL-10, IL-12p70, IL-17A CCL2 (MCP-1) CXCL10 (IP-10), IFN-γ, TGF-β1, and TNF-α. The assay was performed following the manufacturer’s protocol; briefly, samples were diluted 2-fold with assay buffer and 50 mL of sample or standard was mixed with 25 μL of previously vortexed mixed beads in a V-bottom plate, then incubated for 2 h, centrifuged at 250 rpm for 5 min, and washed two times with 200 μL wash buffer, 25 μL of detection antibody were added and incubated as above, and without washing, 25 μL of SA-PE were added and incubated for 30 min, and afterward, centrifugation and washing steps were repeated, adding a final 150 μL of wash buffer to each well before being individually transferred to microtubes for the acquisition in a BD FACS AriaTM Fusion (BD Biosciences, San Jose, CA, USA). Analytes concentrations were calculated using the LEGENDplex Data Analysis SoftwareCatalog 740932 Version 9. Software GraphPad Prism version 9.4.0.

### 4.10. Statistical Analysis

Continuous variables are expressed as mean ± standard deviation or median with the minimum and maximum values. Categorical variables, such as frequencies and percentages, are reported. Normality distribution was evaluated using the Shapiro–France test. Non-parametric tests (Mann–Whitney) or Student’s tests, according to the Gaussian distribution, were performed to detect significant independent variables.

Some variables were standardized and Bonferroni correction was adjusted for multiple comparisons. For the paired analyses (before–after), the Friedman or Wilcoxon test was used with the signed-rank test according to the distribution of the data. Pearson’s Chi-square (χ^2^) test or Fisher’s exact test were used to compare proportions between two groups. For the multivariate analysis with the confounding factors, binary logistic regression was used. For time and group analyses, repeated-samples analysis and panel data tests of different models (pooled model, model for longitudinal data, marginal approximation model, and multilevel model) were performed. A general adjustment was made to select by random age using propensity matching between treated and untreated patients.

We perform a canonical correlation analysis (CC) to analyze the correlation of two subsets of variables. In the first set, we include the x expressed as U (p) (X1, X2, …. XP) in which the variables that they made up were (IL-1β, IL-2, IL4, IL-6, IL-8, IL-10, IL-12p70, IL-17A, TNF-α, INFγ, IP-10, MCP-1, and TGFβ-1). In the second set of variables, expressed by V(q) (Y1, Y2, …. Yq). We included the SOFA score, C-reactive protein, procalcitonin, lipoperoxidation, selenium, thiols, vitamin C, carbonylation, glutathione, Gpx, GST, GR, SOD, and antioxidant capacity (CAT). We include more than two variables on both sides of the equation in this type of analysis. The main objective of CC is to identify the linear combinations and the value of the response of that correlation.

U1 ¼ a11X1 þ a12X2 þ…… þ a1pXp

U2 ¼ a21X1 þ a22X2 þ…… þ a2pXp

Ur ¼ ar1X1 þ ar2X2 þ…… þ arpXp

And with the predictive variable, it is expressed as follows:

V1 ¼ b11Y1 þ b12Y2 þ…… þ b1pYp

V2 ¼ b21Y1 þ b22Y2 þ…… þ b2pYp

Vr ¼ br1Y1 þ br2Y2 þ…… þ brpYp

The largest correlation was identified between U1 and V1; the second largest correlation was between U2 and V2, as long as there was no correlation between U1 and U2, nor between V1 and V2; and the third largest correlation was between U3 and V3 and, as described above. There could be no alteration between U3, and U1 and U2, nor between V3, and V1 and V2. These combinations are known as canonical variables. Differences were considered statistically significant when the *p*-value was <0.05. Statistical analyses were performed using the STATA V.16 Software and Sigma Software Plot 14 (Jendel Corporation, 1986–2017, New York, NY, USA).

### 4.11. Ethical Aspects

A signed informed consent form was obtained from each participant in accordance with the Declaration of Helsinki, as amended at the Congress in Tokyo, Japan. This research was approved by the Ethics, Biosafety and Research Committees of the National Institute of Cardiology (INCICh registry number: PT 10-0-76) and the ABC Campus Observatory Medical Center, approval number ABC-18-19; Registry of Trial: Clinical-Trials.gov Identifier: NCT 03557229.

## 5. Conclusions

Patients treated with antioxidants such as Vit C, Vit E, NAC, or MT, in addition to standard therapy, show reduced levels of proinflammatory cytokines and improved regulatory function in Il-2, IL-12, and IFN. There is improvement of the antioxidant capacity and reduction in biomarkers of oxidative stress with evidence of decreased organ damage measured by SOFA score. Combining antioxidants such as Vit C, MT, and NAC associated with standard therapy is a potential perspective in investigation through randomized clinical trials since it could improve other clinical conditions and pathophysiological mechanisms that converge in septic shock. Regarding the damage mechanisms involved, there is no evidence of improvement with the use of anticytokine biologicals and anti-apoptosis therapy, which evidences the need of research studies to follow for the comprehensive management of sepsis.

## Figures and Tables

**Table 1:** Demographic characteristics of patients in each group of treatment.

	Vit C *n* = 25	Vit E *n* = 27	NAC *n* = 24	MT. *n* = 26	Control *n* = 29	*p*
Age	62 (58–78)	70 (51–77)	68.5 (58.5–78)	62.5 (58–69)	75 (65–81)	0.10
Weight, kg	70 (65–80)	71 (60–82)	69.5 (56.5–80)	66 (60–78)	70 (61–80)	0.80
Height	1.7 (1.5–1.7)	1.7 (1.6–1.7)	1.7 (1.6–1.8)	1.7 (1.6–1.71)	1.7 (1.6–1.7)	0.39
Body mass index	24.9 (23–30.1)	24.6 (22.8–29)	23 (20.7–6.1)	25.35 (21–28)	25 (23.4–28.6)	0.41
Male/Female	10 (40)/15 (60)	17 (62.96)/10 (37.04)	14 (58.33)/10 (41.67)	13 (50)/13 (50)	16 (55.17)/13 (44.83)	0.53 §
SAPSII	39.44 ± 14.10	45.70 ± 16.37	42.41 ± 19.84	40.88 ± 16.80	47.20 ± 7.11	0.60 ¥
APACHEII	14 (12–19)	20 (15–24)	15.5 (11–20.5)	16.5 (10–21)	17 (15–25)	0.15
SOFA	8 (6–9)	9 (7–11)	8 (4–10)	8 (6–9)	9 (7–11)	0.42
NUTRIC	4.16 ± 2.21	4.81 ± 1.64	4.08 ± 1.83	3.88 ± 1.79	5.10 ± 1.54	0.41 ¥
Diabetes mellitus	7 (28.00)	5 (18.52)	5 (20.83)	6 (23.08)	8 (27.59)	0.90 *
Arterial hypertension	10 (40.00)	11 (40.74)	12 (50.00)	8 (30.77)	15 (51.72)	0.53 §
COPD	1 (4.00)	5 (18.52)	4 (16.67)	2 (7.69)	0 (0.00)	0.05 *
Smoking	17 (68.00)	12 (44.44)	9 (37.50)	15 (57.69)	14 (48.28)	0.22 §
Cancer	6 (24.00)	11 (40.74)	8 (33.33)	8 (30.77)	14 (48.28)	0.39 §
Cirrhosis	2 (8.00)	2 (7.41)	1 (4.17)	1 (3.85)	4 (13.79)	0.71 *
CKD	2 (8.00)	3 (11.11)	4 (16.67)	3 (11.54)	3 (10.34)	0.92 *
Hypothyroidism	4 (16.00)	4 (14.81)	2 (8.33)	6 (23.08)	7 (24.14)	0.56 *
CVD	3 (12.00)	0 (0.00)	1 (4.17)	2 (7.69)	3 (10.34)	0.41 *
Heart stroke	1 (4.00)	0 (0.00)	3 (12.50)	2 (7.69)	2 (6.90)	0.43 *
Atrial fibrillation	3 (12.00)	2 (7.41)	3 (12.50)	5 (19.23)	4 (13.79)	0.79 *
DVT	0 (0.00)	0 (0.00)	1 (4.17)	0 (0.00)	2 (6.90)	0.39 *
PE	0 (0.00)	0 (0.00)	2 (8.33)	1 (3.85)	2 (6.90)	0.42 *

Vit C: vitamin C; Vit E: vitamin E; NAC: N-acetylcysteine; MT: melatonin; TX: treatment; COPD: chronic obstructive pulmonary disease; CKD: chronic kidney disease; CVD: cerebral vascular dis-ease; DVT: Deep venous thrombosis; PE: Pulmonary embolism. Values are expressed as median (p25–p75); Statistical test: Kruskal–Wallis test, ¥ one-way ANOVA, § chi-squared test, and * Fisher’s exact test.

**Table 2:** Demographic characteristics of patients according to treatment group.

Characteristics	Vit C *n* = 25	Vit E *n* = 27	NAC *n* = 24	MT. *n* = 26	Control *n* = 29	*p*
Reason for admission, *n* (%)						
Surgical	7 (28.00)	6 (22.22)	5 (20.83)	4 (15.38)	13 (44.83)	0.14
Non-surgical	18 (72.00)	21 (77.78)	19 (79.17)	22 (84.62)	16 (55.17)	
Place of admission, *n* (%)						
Emergency	14 (58.33)	15 (55.56)	13 (54.17)	18 (69.23)	14 (48.28)	0.76
Operating room	4 (16.67)	5 (18.52)	3 (12.50)	3 (11.54)	7 (24.14)	
Hospitalization	4 (16.67)	7 (25.93)	8 (33.33)	5 (19.23)	7 (24.14)	
Diagnoses at admission, *n* (%)						
Cardiovascular	0 (0.00)	0 (0.00)	0 (0.00)	2 (7.69)	2 (6.90)	0.82
Respiratory	6 (25.00)	6 (22.22)	7 (29.17)	4 (15.38)	5 (17.24)	
Gastrointestinal	9 (37.50)	5 (18.52)	4 (16.67)	5 (19.23)	6 (20.69)	
Neurological	1 (4.17)	1 (3.70)	1 (4.17)	2 (7.69)	2 (6.90)	
Sepsis	6 (25.00)	1 (3.70)	7 (29.17)	9 (34.62)	12 (41.38)	
Trauma	0 (0.00)	1 (3.70)	1 (4.17)	0 (0.00)	0 (0.00)	
Metabolic	1 (4.17)	1 (3.70)	0 (0.00)	0 (0.00)	1 (3.45)	
Hematologic	0 (0.00)	2 (7.41)	0 (0.00)	1 (3.85)	1 (3.45)	
Renal/Genitourinary	0 (0.00)	1 (3.70)	3 (12.50)	3 (11.54)	0 (0.00)	
Site of infection, *n* (%)						
Pulmonary	7 (29.17)	11 (40.74)	9 (39.13)	11 (42.31)	10 (34.48)	0.83
Gastrointestinal	10 (41.67)	8 (29.63)	5 (21.74)	5 (19.23)	11 (37.93)	
Nefrourinary	3 (12.50)	3 (11.11)	6 (26.09)	6 (23.08)	3 (10.34)	
CNS	0 (0.00)	2 (7.41)	0 (0.00)	0 (0.00)	1 (3.45)	
Skin and soft tissues	2 (8.33)	2 (7.41)	2 (8.70)	2 (7.69)	2 (6.90)	
Endocarditis	0 (0.00)	0 (0.00)	0 (0.00)	0 (0.00)	1 (3.45)	
Gastrointestinal	0 (0.00)	1 (3.70)	0 (0.00)	2 (7.69)	1 (3.45)	

Vit C: vitamin C; Vit E: vitamin E; NAC: N-acetylcysteine; MT: melatonin.

**Table 3:** Levels of cytokines in patients from the different treatment groups.

	Proinflammatory			Anti-Inflammatory		
	Before	After		Before	After	
Group	IL-1β	IL-1β	*p*	IL-4	IL-4	*p*
Vit C	9.8 (8.12–5700)	8.1 (6.4–178.79)	0.16	3.3 (0.39–328)	2.2 (0.39–65.2)	0.53
Vit E	8.1 (8.1–360)	8.1 (8.1–170.7)	0.66	5.35 (0.39–275.5)	13.7 (0.39–484.9)	0.21
NAC	10.8 (8.1–139.5)	8.1 (8.1–172.7)	0.27	4.05 (0.39–4262.7)	6.4 (0.39–844.4)	0.39
MT	8.1 (8.1–691)	8.1 (7.1–1050)	0.34	3.8 (0.39–184)	2.09 (0.39–117)	0.04
Control	8.1 (8.1–4110)	8.2 (8.1–1337)	0.28	3.7 (0.39–1307.7)	1.5 (0.39–1000)	0.52
	IL-6	IL-6		IL-10	IL-10	*p*
Vit C	124.89 (22.35–546.49)	59.82 (14.93–148.1)	0.006	73.3 (2.97–1094.30)	66 (2.97–2762.5)	0.56
Vit E	106.345 (5.64–512.885)	16.965 (0.94–233.24)	0.07	106.3 (2.9–1322)	74 (2.9–362.9)	0.17
NAC	127.48 (31.27–997.595)	69.85 (3.805–231.895)	0.90	56.5 (4.6–1007.97)	54.7 (7.34–1370.7)	0.88
MT	344.87 (15.13–564.88)	23.25 (7.45–238.45)	0.001	70.5 (6.7–1815)	57.4 (4.1–5.6)	0.11
Control	236.24 (22.92–2197.01)	35.97 (6.91–112.95)	0.002	107 (2.97–1683)	55.1 (1.9–3960)	0.08
	IL-8	IL-8	*p*	TGF-β	TGF-β	*p*
Vit C	12.6 (1.45–83.6)	6.1 (1.45–46.34)	0.14	44.7 (8.8–414.2)	46.9 (0–535.2)	0.80
Vit E	19.05 (1.45–241)	8.6 (1.45–269)	0.16	63 (8.8–452.8)	52.9 (0–826.7)	0.41
NAC	14.8 (1.45–283)	9.5 (1.84–269.9)	0.73	27.6 (8.8–411.6)	52.9 (8.8–789.7)	0.08
MT	13.8 (1.45–227.17)	8.9 (1.45–367)	0.001	45.09 (8.8–616.3)	51.4 (8.8–359.06)	0.53
Control	21.9 (1.45–688.8)	7.6 (1.45–100)	0.001	45.1 (8.8–1033)	75.8 (8.8–356.9)	0.67
	Proinflammatory			Regulatory cytokines		
	TNF-α	TNF-α		IL-2	IL-2	*p*
Vit C	0.80 (0.41–99.6)	0.65 (0.41–18.9)	0.26	1.4 (0.45–100.4)	0.45 (0.45–10.6)	0.36
Vit E	0.74 (0.41–119)	0.8 (0.41–144.9)	0.85	0.56 (0.30–51.4)	1.13 (0.45–102.7)	0.01
NAC	1.06 (0.41–76.6)	0.1 (0.41–144.9)	0.04	0.58 (0.45–51.4)	0.66 (0.45–102.7)	0.15
MT	0.64 (0.41–20.4)	0.80 (0.41–49.6)	0.84	0.45 (0.45–8.9)	0.45 (0.45–18.4)	0.34
Control	0.41 (0.41–32.6)	0.54 (0.41–65)	0.79	0.51 (0.45–3.80)	0.45 (0.45–15.3)	0.54
	IP-10	IP-10	*p*	IL-12	IL-12	*p*
Vit C	73.3 (2.97–1094.3)	66.05 (2.97–2762.53)	0.57	1.2 (0.85–21.8)	1.06 (0.85–32.1)	0.12
Vit E	106.3 (2.97–1322.2)	74.5 (2.9–362.9)	0.17	1.49 (0.85–176.4)	1.45 (0.85–222.7)	0.01
NAC	56.5 (4.68–1007.9)	54.7 (7.34–1370.7)	0.88	1.49 (0.85–62.36)	1.4 (0.85–187.02)	0.04
MT	69.6 (6.7–1001.7)	57.4 (4.1–565.8)	0.11	1.1 (0.85–27.8)	0.92 (0.85–49.1)	0.11
Control	107.6 (2.9–1683.8)	55.1 (1.9–3960.96)	0.08	1.2 (0.85–46.7)	1.2 (0.85–59.4)	0.75
	MCP-1	MCP-1	*p*	IFN-γ	IFN-γ	*p*
Vit C	324 (15.1–4321)	216 (5.3–1641)	0.11	3.08 (3.05–31.40)	3.05 (3.05–23.01)	0.46
Vit E	189.1 (1.6–2700)	119 (1.6–2866)	0.45	3.15 (3.05–20.77)	3.05 (3.05–96.16)	0.03
NAC	257.6 (78.08–2954.7)	152.8 (23.5–2569.9)	0.44	3.49 (3.05–23.98)	3.93 (3.05–36.71)	0.15
MT	295 (23.2–3479)	210.7 (31.2–2504.4)	0.01	3.05 (3.05–18.18)	3.05 (3.05–14.09)	0.53
Control	316 (38–4110)	185. (11.03–1000)	0.002	3.2 (3.05–67.44)	3.05 (3.05–33.24)	0.21

Vit C: vitamin C. Vit E: vitamin E. NAC: N-acetylcysteine. MT: melatonin. MCP-1: chemotactic protein from monocytes 1: range test signaled by Wilcoxon. Values are expressed in pg/mL.

**Table 4:** Canonical correlation analysis between cases and controls with septic shock pretreatment.

Variable	Level	Variable	Coef.	Std. Err.	t	*p*-Value	IC95%		Corr.
u1	High	PCT	0.0172182	0.0042318	4.07	0.0001	0.0085873	0.0258491	0.9529
		Carbonyl	−0.0068545	0.0030781	−2.23	0.033	−0.0131324	−0.0005766	
	Low	TAC	−0.0002907	0.0000967	−3.01	0.005	−0.000488	−0.0000935	
		GSH	5.295229	1.522535	3.48	0.002	2.189998	8.400459	
		Vit C	6.144225	0.6809717	9.02	0.001	4.755374	7.533075	
v1	Low	IL4	−0.0073968	0.0025368	−2.92	0.007	−0.0125706	−0.002223	
	High	IP10	0.0009865	0.0001994	4.95	0.001	0.0005798	0.0013932	
		IL1B	0.0002558	0.000123	2.08	0.046	5.01 × 10^−6^	0.0005067	
		MCP 1	−0.0006084	0.0001506	−4.04	0.001	−0.0009155	−0.0003012	
		IL6	0.0003399	0.0000436	7.8	0.001	0.0002511	0.0004288	
		ILl17a	−0.0098983	0.0025994	−3.81	0.001	−0.0151998	−0.0045967	
		TNFα	0.049159	0.0144382	3.4	0.002	0.0197122	0.0786059	
u2	High	LPO	−0.1764843	0.0744333	−2.37	0.024	−0.328292	−0.0246766	0.8643
		Carbonyl	0.0164766	0.0056307	2.93	0.006	0.0049928	0.0279604	
	Low	GSH	−11.67688	2.785091	−4.19	0.001	−17.35711	−5.996647	
		Selenium	492.1174	139.0012	3.54	0.001	208.6226	775.6122	
		Thiol	−0.1522415	0.0385513	−3.95	0.001	−0.2308675	−0.0736155	
v2	Low	il4	0.0124842	0.0046404	2.69	0.011	0.00302	0.0219484	
		IL12p70	0.0367106	0.0155704	2.36	0.025	0.0049544	0.0684667	
		IFNy	−0.0140323	0.0035662	−3.93	0.001	−0.0213055	−0.006759	
		TGFB1	0.0031482	0.00154	2.04	0.049	7.40 × 10^−6^	0.006289	
	High	IL1B	−0.0007156	0.000225	−3.18	0.003	−0.0011744	−0.0002568	
		MCP1	0.0013014	0.0002755	4.72	0.001	0.0007396	0.0018632	
		IL6a	0.0002323	0.0000797	2.92	0.007	0.0000698	0.0003947	
u3	High	SOFA	−0.3846204	0.1060993	−3.63	0.001	−0.6010113	−0.1682295	0.8218
		PCT	−0.0219292	0.009223	−2.38	0.024	−0.0407397	−0.0031188	
		PCR	0.0441247	0.0167844	2.63	0.013	0.0098926	0.0783568	
	Low	GSH	6.547262	3.318249	1.97	0.057	−0.2203513	13.31488	
		GSHpx	−6.29239	1.747836	−3.6	0.001	−9.857125	−2.727655	
v3	Low	IL4	0.0070789	0.0055288	1.28	0.21	−0.004197	0.0183549	
		TGFB1	0.0038936	0.0018348	2.12	0.042	0.0001515	0.0076356	
		IL12p70	−0.0400625	0.0185511	−2.16	0.039	−0.0778978	−0.0022272	
	High	IL1B	−0.0007372	0.000268	−2.75	0.01	−0.0012838	−0.0001905	
		IL8	0.0300677	0.0085392	3.52	0.001	0.0126518	0.0474836	

The canonical correlation revealed a significantly high correlation between the first (0.9529), the second (0.8643), and third (0.8218). Lambda test with an F = 0.001; the Lawley–Hotteling trace and Loy’s largest root yielded the same statistical significance.

## Data Availability

Data are contained within the article and Supplementary Materials.

## Supplementary Materials

The supporting information can be downloaded at: https://www.mdpi.com/article/10.3390/ijms242316610/s1. References [126,127,128,129,130,131,132,133] are cited in the supplementary materials.
