# Supplementary material for: Impact of Treatment with Antioxidants as an Adjuvant to Standard Therapy in Patients with Septic Shock: Analysis of the Correlation between Cytokine Storm and Oxidative Stress and Therapeutic Effects
Source: Int J Mol Sci. 2023 Nov 22;24(23):16610. doi: 10.3390/ijms242316610 (PMC10706209; doi:10.3390/ijms242316610)
Supplement: Supplementary file 1 [file ijms-24-16610-s001.zip › Supplement S1.pdf]

Sequential Organ Failure Assessment (SOFA) between antioxidant treatment and control groups. Results of group analysis via one-way ANOVA and repeated measures between times is shown.

	**Day 0**	**Day 1**	**Day2**	**Day3**	**Day 4**	**Day 5**	**Repeated measures p-value**
Vit C	8 (6-9)	5 (4-10)	2 (2-9)	3.5 (2-6)	2.5 (1-5.5)	3 (1-5)	0.001
Vit E	9 (7-12)	8 (7-12)	7 (5-11)	6 (4-10)	5 (3-8)	5 (2-7)	0.522
NAC	7 (6-10)	7.5 (7-10)	7 (4.5-8.5)	6 (3.5-8.5)	5 (2-8)	4 (2-7)	0.003
MT	8 (6-10)	7 (5-9)	7 (5-7)	4 (2-5)	4 (2-5)	2 (1-5)	0.001
Control	9 (7-11)	9 (7-11)	7 (5-9)	7 (5-10)	6 (4-7.5)	6 (4-7)	0.10
**One-way ANOVA p-value**	0.287	0.053	0.179	0.023	0.04	0.01	

Group	Regression coefficient	SE.	95% CI	p-value
Vit C	-2.303	0.430	[-3.14- 1.4]	0.001
Vit E	-0.262	0.409	[-1.06 a 0.54]	0.522
NAC	-0.891	0.419	[-1.71 a -0.06]	0.003
MT	-1.745	0.414	[-2.55 a – 0.93]	0.001
Control	0	Base		

Repeated-measures statistical analysis was used to analyze change over time by treatment group: SOFA (Sequential Organ Failure Assessment) score was assessed from day 0 to day 5 in each group and was analyzed using the marginal approximation model of the repeated-measures statistics. In the lower part of the table, the results of the marginal approximation regression model which obtain statistical significance are shown. Comparison between groups was performed using one-way ANOVA. Vit C: Vitamin C; Vit E: vitamin E; NAC: N-acetylcysteine; MT:melatonin; CI:confidence interval; SE= standard error
