# Supplementary material for: Impact of Treatment with Antioxidants as an Adjuvant to Standard Therapy in Patients with Septic Shock: Analysis of the Correlation between Cytokine Storm and Oxidative Stress and Therapeutic Effects
Source: Int J Mol Sci. 2023 Nov 22;24(23):16610. doi: 10.3390/ijms242316610 (PMC10706209; doi:10.3390/ijms242316610)
Supplement: Supplementary file 1 [file ijms-24-16610-s001.zip › Supplement S2.pdf]

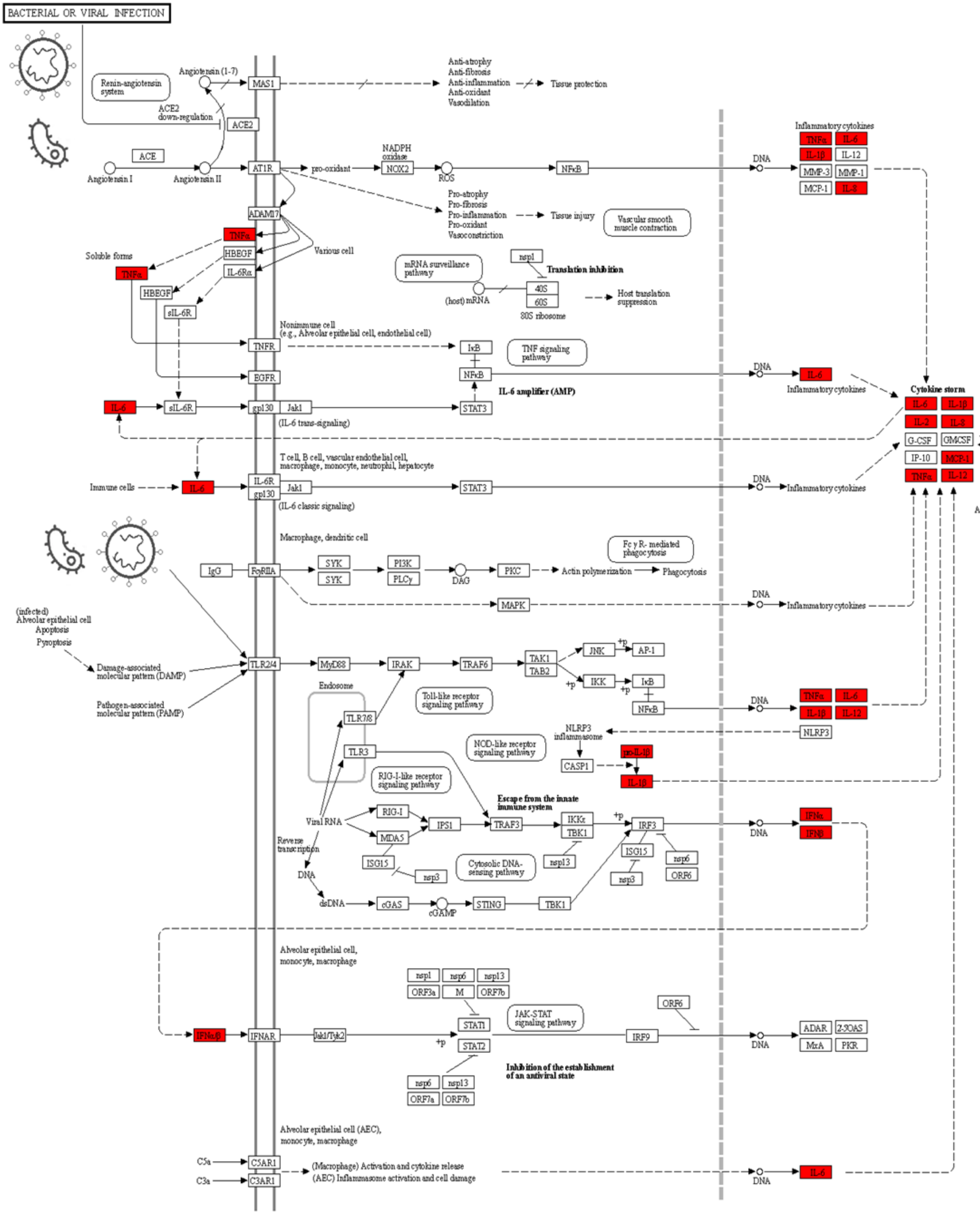
BACTERIAL OR VIRAL INFECTION
Renin-angiotensin system
Angiotensin (1-7)
MAS1
Anti-atrophy
Anti-fibrosis
Anti-inflammation
Anti-oxidant
Vasodilation
Tissue protection
ACE2 down-regulation
ACE2
ACE
Angiotensin I
Angiotensin II
AT1R
pro-oxidant
NADPH oxidase
NOX2
ROS
NFκB
DNA
Inflammatory cytokines
TNFα
IL-6
IL-1β
IL-12
MMP-3
MMP-1
MCP-1
IL-8
ADAM17
Pro-atrophy
Pro-fibrosis
Pro-inflammation
Pro-oxidant
Vasoconstriction
Tissue injury
Vascular smooth muscle contraction
Soluble forms
TNFα
HBEGF
IL-6Rα
Various cell
nsp1
mRNA surveillance pathway
Translation inhibition
40S
60S
(host) mRNA
80S ribosome
Host translation suppression
sIL-6R
Nonimmune cell
(e.g., Alveolar epithelial cell, endothelial cell)
TNFR
IκB
TNF signaling pathway
EGFR
NFκB
IL-6 amplifier (AMP)
IL-6
gp130
Jak1
STAT3
(IL-6 trans-signaling)
Cytokine storm
IL-6
IL-1β
IL-2
IL-8
G-CSF
GMCSF
IP-10
MCP-1
TNFα
IL-12
T cell, B cell, vascular endothelial cell, macrophage, monocyte, neutrophil, hepatocyte
Immune cells
IL-6R
(IL-6 classic signaling)
Macrophage, dendritic cell
Fc γ R- mediated phagocytosis
IgG
FcγRIIA
SYK
PI3K
PLCγ
DAG
PKC
Actin polymerization
Phagocytosis
MAPK
(infected)
Alveolar epithelial cell
Apoptosis
Pyroptosis
Damage-associated molecular pattern (DAMP)
Pathogen-associated molecular pattern (PAMP)
TLR2/4
MyD88
IRAK
TRAF6
TAK1
TAB2
JNK
AP-1
IKK
+p
Endosome
TLR7/8
Toll-like receptor signaling pathway
TLR3
NLRP3 inflammasome
NLRP3
NOD-like receptor signaling pathway
CASP1
pro-IL-1β
IL-1β
RIG-I-like receptor signaling pathway
Escape from the innate immune system
Viral RNA
RIG-I
MDA5
IPS1
TRAF3
IKKε
TBK1
IRF3
ISG15
nsp6
ORF6
nsp13
nsp3
IFNα
IFNβ
Reverse transcription
Cytosolic DNA-sensing pathway
dsDNA
cGAS
cGAMP
STING
Alveolar epithelial cell, monocyte, macrophage
nsp1
ORF3a
M
ORF7b
JAK-STAT signaling pathway
ORF6
IFNα,β
IFNAR
Jak1/Tyk2
STAT1
STAT2
IRF9
ADAR
2-5OAS
MxA
PKR
Inhibition of the establishment of an antiviral state
nsp6
nsp13
ORF7a
ORF7b
Alveolar epithelial cell (AEC), monocyte, macrophage
C5a
C5AR1
C3a
C3AR1
(Macrophage) Activation and cytokine release
(AEC) Inflammasome activation and cell damage

Supplement 2 The main signaling pathways activated during the development of septic shock,which can be triggered by the exposure to viral or bacterial infection. The renin–angiotensin system, the classic pathway of activation, and the lectin pathway are illustrated. The interleukins evaluated in this study are shown in red, and the possible interactions that may occur within these pathways are shown. The key clinical manifestations associated with organ damage are also depicted. In the renin-angiotensin system, in the presence of any infection (bacteria, fungi, or viruses), the ATIR receptor is activated, with a prooxidant response through ROS that triggers the NFkB transcription factor. Through second messengers, this factor participates in the transcription of inflammatory interleukins, which ends in a cytokine storm. This work shows an increase in interleukins (IL6 TNFa, IL1, IL8 and MCP1). The cytokine storm triggers the activation of macrophages and neutrophils, favoring ROS and NO accumulation. These mechanisms evolve to multiple organ failure in septic shock. The ATIR receptor in the cell membrane can also activate the ADAM17 protein, activating the TNF signaling pathway through TNFα and Jak1. These factors induce IL6 overexpression, generating a cytokine storm and leading to failure. Organic. The TLR2/4 membrane protein, which, through the NFkβ transcription factor, activates the overexpression of TNFa, IL6, and IL1, and this cycle also ends in an increase in cytokines and organ failure in sepsis. The pathways that trigger dysregulated oxidant stress and inflammation converge through common signaling pathways. In red, we show the increased interleukins in this work, leading to organ failure.
