# Supplementary material for: Impact of Treatment with Antioxidants as an Adjuvant to Standard Therapy in Patients with Septic Shock: Analysis of the Correlation between Cytokine Storm and Oxidative Stress and Therapeutic Effects
Source: Int J Mol Sci. 2023 Nov 22;24(23):16610. doi: 10.3390/ijms242316610 (PMC10706209; doi:10.3390/ijms242316610)
Supplement: Supplementary file 1 [file ijms-24-16610-s001.zip › Supplement S4.pdf]

METHODOLOGY FOR THE USE AND APPLICATION OF ANTIOXIDANTS

The Pharmacotherapeutic Follow-up of the antioxidants used in the clinical trial had Strict surveillance of the administration of medications by the CMABC Pharmacy Department to establish that antioxidants were administered as much as possible under the same conditions, both in the preparation and preparation administration hours. The Pharmacobiologists considered the appropriate need to administer the drugs according to the pharmacokinetics, interactions, and the type of enteral diet that is commonly used in critical ICU patients.

The management, acquisition, storage, dispensing, preparation, handling, administration, and pharmacological interactions of the drugs used in the study were carried out by the CMAB Pharmacy Department, following the policies established by the institution. Next, we describe each of these items.

Choice of medicines (management, acquisition, protection).

1. Medications containing only the active principle of interest were selected: N-acetylcysteine, Vitamin C, Vitamin E and Melatonin, taking into account the following requirements: to. Medicines that have a health registry.

b. No herbal products or products containing any other active ingredient.

c. Medications with immediate release pharmaceutical technology.

d. It will have a national supply.

2. The selected drugs were:

Commercial drugs selected for the study.				
Active principle	Tradename	Dose	Pharmaceutical Technology	Health Register
N-acetyl cysteine	Lysomucil (1)	600 mg	effervescent tablet	506M99, SSA IV
Vitamin C	Redoxon (2)	1000 mg	effervescent tablet	62535 SSA VI
Vitamin E	Eternal (3)	400 international units (400 mg)	soft gelatin capsules	81210 SSA

Melatonin	Benedorm (4)	5 mg	sublingual tablet	389M98, SSA

1. Once the drugs were selected, the CMABC Pharmacy department managed and acquired the total doses of the study to guarantee that they belonged to the same manufacturing batch; they were stored in said department under the pertinent conditions for stability and were conditioned for dispensing as a unit dose.
2. To guarantee the administration and absorption of the drugs, an information search was carried out to identify the recommendations for drug administration both orally and by tube (for patients under sedation).
3. Se realizaron pruebas de la administración de los fármacos por sonda enteral y se generaron una serie de recomendaciones para cada uno, como se describe más adelante. Drug administration tests were carried out by enteral tube, and a series of recommendations were generated for each one, as described below.

Dispensing

4. Once the patient was included, the Pharmacy department was notified, which was responsible for providing each required administration in a unit dose.
5. Nursing staff were also instructed to administer said medications, so that they were handled under the same conditions.

Administration

Total dose : N-Acetylcysteine, 1200 mg, orally/gavage, every 12 hours, for five days

6. Oral route or via nasogastric tube: A 600 mg N-Acetylcysteine tablet was dissolved in 30 ml of water and administered immediately; then, the glass was rinsed with 10 ml of water and administered to the patient.
7. In the case of administration by nasogastric tube, a 60 ml syringe was used, which was transferred from the vessel and injected directly into the box, performing the same rinsing process.

Total dose : Melatonin, 50 mg, orally/gavage, every 24 hours, for 5 days

We were recommended to carry out the preparation, an extemporaneous suspension from the commercially available sublingual tablets, since we did not have a pharmaceutical form that contained the dose used in the study (50 mg); Said formula was extracted from the methodology described by Johnson CE, et al in Stability of an extemporaneous alcohol-free melatonin suspension; that guarantees a stability of up to 90 days (5).

Total dose: Vitamin C, 1 g, orally/gavage, every 6 hours, for 5 days

8. Oral route / via nasogastric tube: A 1000 mg vitamin C tablet was dissolved in 30 ml of water and administered immediately, rinsing the glass with 10 ml of water and administered to the patient.
9. In the case of administration by nasogastric tube, a 60 ml syringe was used, with which it was transferred from the glass and injected directly into the box, performing the same rinsing process.t.
10. At all times protect from light, since it is photosensitive.

Vitamin E, 1200 mg (International Units), orally / via tube, every 24 hours, for 5 days

- Preparation of vitamin E capsules 400 international units by nasogastric tube.
- to. Material
- 1 syringe of 60 ml.
- 1 TEC stopper for syringe.
- 30 ml of hot water.
- 3 Eternal Capsules (Vitamin E) 400 International Units.
    a. Instructions
        i. Yo. Withdraw the plunger from the syringe.
        ii. ii. Place TEC stopper on the syringe.
        iii. iii. Deposit the 3 Eternal (Vitamin E) capsules inside the syringe.
        iv. iv. Add 20 ml of hot water, between 30-40 °C (86-104 °F) [Vitamin E boiling point, 662 °F at 760 mmHg (6)].

v. v. Strain the plunger of the syringe.
vi. saw. Shake vigorously until the capsules dissolve.
vii. vii. Remove the TEC cap from the syringe.
viii. viii. Assess the temperature of the mixture and administer as soon as possible.
ix. ix. Add 10 mL of hot water to the syringe, shake vigorously..
x. . Assess temperature and administer as soon as possible.

11. It was taken care that the administration of vitamin E was always carried out during or after food since its absorption depends on the presence of pancreatic enzymes . (7,8).

Drug interactions

12. The detection and management of drug interactions was carried out using the Micromedex database, in the opinion of the pharmacist in charge and in conjunction with the medical and nursing team according to the diagram 1.

Diagram1

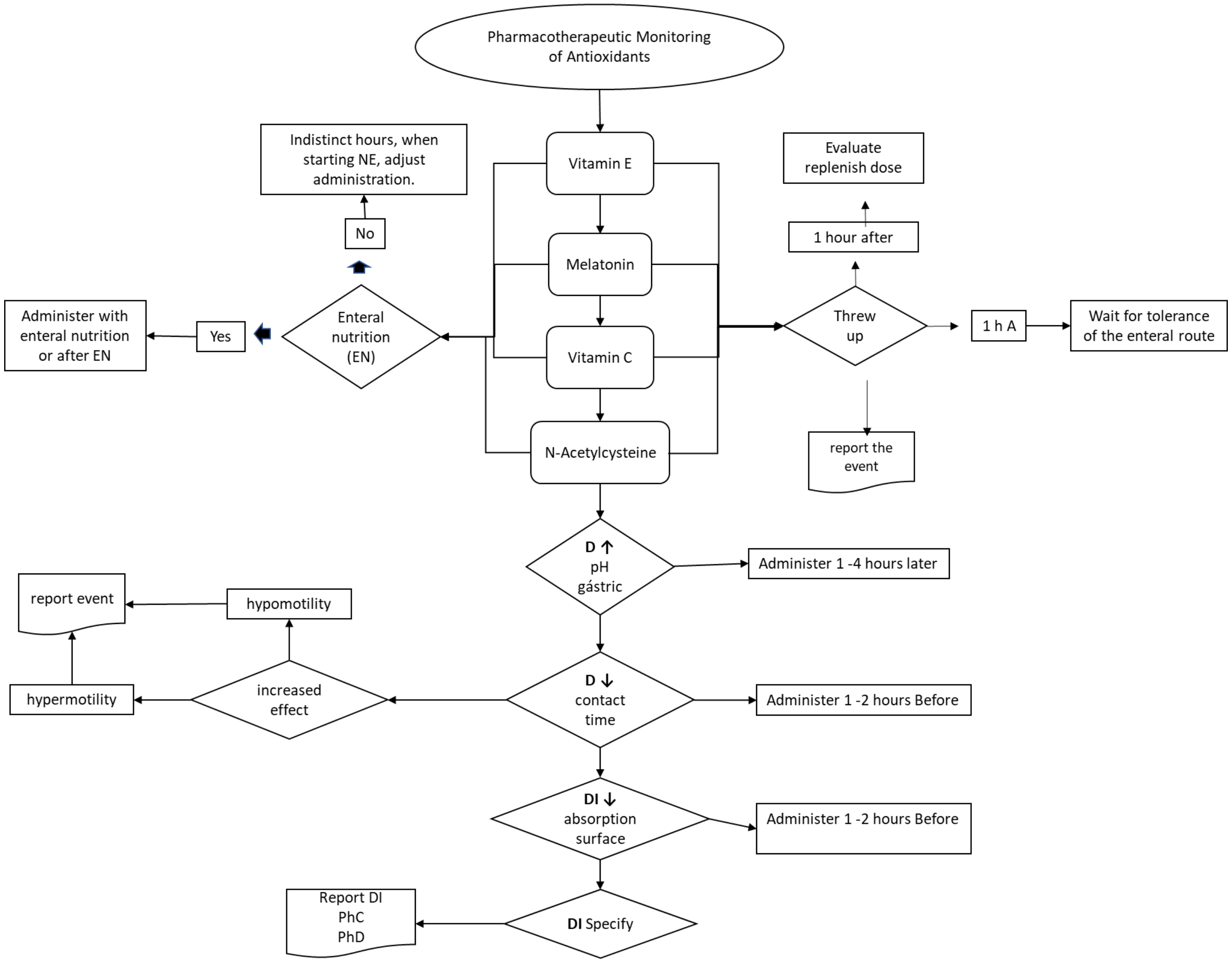

Pharmacotherapeutic Monitoring of Antioxidants
Vitamin E
Melatonin
Vitamin C
N-Acetylcysteine
Indistinct hours, when starting NE, adjust administration.
No
Enteral nutrition (EN)
Yes
Administer with enteral nutrition or after EN
Evaluate replenish dose
1 hour after
Threw up
1 h A
Wait for tolerance of the enteral route
report the event
D ↑ pH gástric
Administer 1 -4 hours later
D ↓ contact time
Administer 1 -2 hours Before
increased effect
hypomotility
hypermotility
report event
DI ↓ absorption surface
Administer 1 -2 hours Before
DI Specify
Report DI PhC PhD

Diagram

Diagram 1. Pharmacotherapeutic monitoring of the antioxidants used in the clinical trial. The figure shows the considerations made by the Department of Pharmacy of the Medical Center MABC to establish that the antioxidants used in the study are administered as much as possible under the same conditions, both in the preparation and administration schedules. Enteral drugs and diets commonly used in critically ill patients in the intensive care unit of the institution were considered. The administration of enteral medicines (N-acetylcysteine, melatonin, vitamin C, and vitamin E) may present alterations in their absorption when used together with drugs that reduce the absorption surface, such as sucralfate (gastroprotective); or that decrease the contact time, such as prokinetics; however, the former can be mitigated by separating the administration of the drugs, considering the absorption site of the antioxidants, the mechanism of action and the place of effect of the gastroprotection; Regarding the prokinetics, it is expected that the negative impact on the absorption of the drugs will be low or null, since they will be used in critically ill patients, who present a decrease in intestinal motility. Attention should only be paid if the patient presents hypermotility and diarrhea, at which point absorption could be decreased. Time intervals (minimum - maximum) were used for the administration of antioxidant agents and drugs. Since the patients could have more drug administrations and enteral nutrition, all the administration schedules were supervised by the pharmacist of the intensive care unit. The events (vomiting, hyper or hypomotility) were documented, in order to assess a decrease or increase in absorption, respectively. Drugs that decrease the absorption surface, eg. sucralfalto (gastroprotective); drugs that decrease contact time, eg. cinitapride (prokinetics); drugs that modify gastric pH, eg. omeprazole (gastroprotective).

Abbreviations: D: Drugs; ex: example, NE: Enteral nutrition, DI: Drug interactions; PhC: Pharmacokinetics, PhD: Pharmacodynamics, ↑: Increases, ↓: Decreases.

Preparación de melatonina suspensión 2.5 mg/ml

Material:

Ora plus 50 ml

Ora sweet 50 ml

Vaso de precipitados, agitador magnético, probeta de 200-250 ml

50 tabletas de melatonina de 5 mg

Instrucciones:

1) Triturar las 50 tabletas de melatonina (Benedorm) de 5 mg (250 mg)
2) Levigar con 50 mL de Ora-plus (Traspasando a un vaso de precipitados con agitador magnético)
3) Poner en agitación suave
4) “Enjuagar” el mortero con 10 mL de Ora-sweet
5) Agregar 30 mL de Ora sweet al vaso donde se está realizando la mezcla
6) Aforar con Ora-sweet cbp 100 mL
7) Cargar 5 jeringas con 20 mL cada una de la solución
8) Colocar tapón tec
9) Colocar etiqueta FL02 con leyenda Melatonina 50 mg/20 mL ADMINISTRACIÓN POR SONDA O VÍA ORAL Fecha límite de uso 90 días, temperatura ambiente. Protegido de la luz (Dispensar en bolsa negra que cubra la bolsa transparente del empaque primario de la preparación).

REFERENCIAS

1. LYSOMUCIL - PLM [Internet]. [cited 2020 Jun 26]. Available from: https://www.medicamentosplm.com/Home/productos/lysomucil_tabletas_efervescentes/42/101/36846/218

2. REDOXON - PLM [Internet]. [cited 2020 Jun 26]. Available from: https://www.medicamentosplm.com/Home/productos/redoxon_tabletas_masticables/

21/101/9525/220

3. ETERNAL CÁPSULAS 400 UI de México [Internet]. [cited 2023 Sep 2]. Available from: https://www.vademecum.es/mexico/medicamento/1280368/eternal+capsulas+400+ui

4. BENEDORM - PLM [Internet]. [cited 2020 Jun 26]. Available from: https://www.medicamentosplm.com/Home/productos/benedorm_tabletas_sublinguales/187/101/10598/224

5. Johnson CE, Cober MP, Thome T, Rouse E. Stability of an extemporaneous alcohol-free melatonin suspension. Am J Heal Pharm. 2011;68(5):420–3.

6. National Center for Biotechnology Information. Vitamin E | C29H50O2 - PubChem. Retrieved May 29, 2022. 2022.

7. Galli F, Azzi A, Birringer M, Cook-Mills JM, Eggersdorfer M, Frank J, et al. Vitamin E: Emerging aspects and new directions. Free Radic Biol Med. 2017;102(September 2016):16–36.

8. Outline C, Concepts A. Distribution in Foods. 2012. 181–211 p.
