# Supplementary material for: Impact of Treatment with Antioxidants as an Adjuvant to Standard Therapy in Patients with Septic Shock: Analysis of the Correlation between Cytokine Storm and Oxidative Stress and Therapeutic Effects
Source: Int J Mol Sci. 2023 Nov 22;24(23):16610. doi: 10.3390/ijms242316610 (PMC10706209; doi:10.3390/ijms242316610)
Supplement: Supplementary file 1 [file ijms-24-16610-s001.zip › Supplement S5.pdf]

Definición detallada de los criterios de puntuación SOFA					
**Sistema**	0	1	2	3	4
**Respiracion PaO2/FiO2, mm Hg (kPa)**	≥400	< 400	< 300	<200 con Apoyo respiratorio	<100 con PaO2/FiO2, mm Hg (kPa) apoyo respiratorio
**Coagulacion Plaquetas, ×103 /µL**	≥150	< 150	<100	<50	<20
**Higado Bilirubina, mg/dL(µmol/L)**	<1.2	1.2–1.9	2.0–5.9	6.0–11.9	>12.0
**Cardiovascular Presion media arterial (PAM) (mm Hg)**	≥ 70mm Hg	< 70mm Hg	Dopamine < 5 or dobutamine (any dose)	Dopamine 5.1–15 or epinephrine ≤ 0.1 or norepinephrine ≤ 0.1	Dopamine > 15 or epinephrine > 0.1 or norepinephrine > 0.1
**Sistema nervioso central Glasgow**	15	13-14	10-12	6-9	<6
**Renal Creatinina, mg/dL (µmol/ L)**	<1.2	1.2–1.9	2.0–3.4	3.5–4.9	>5.0
**Urine output, mL/d**				<500	<200
**SOFA = sequential organ failure assessment**					
**Interpretación del pronóstico de mortalidad según el Score: de 0-1 Mortalidad (0%), 2-3, (6.4%), 4-5 (20.2%), 6-7 (21.5%), 8-9 (33.3 %), 10-11 (50%), 12-14 (95.2%)**					
