# Supplementary figures and images for: Impact of Treatment with Antioxidants as an Adjuvant to Standard Therapy in Patients with Septic Shock: Analysis of the Correlation between Cytokine Storm and Oxidative Stress and Therapeutic Effects
Source: Int J Mol Sci. 2023 Nov 22;24(23):16610. doi: 10.3390/ijms242316610 (PMC10706209; doi:10.3390/ijms242316610)

**Supplement 1. Screening, Randomization and Outcomes.**

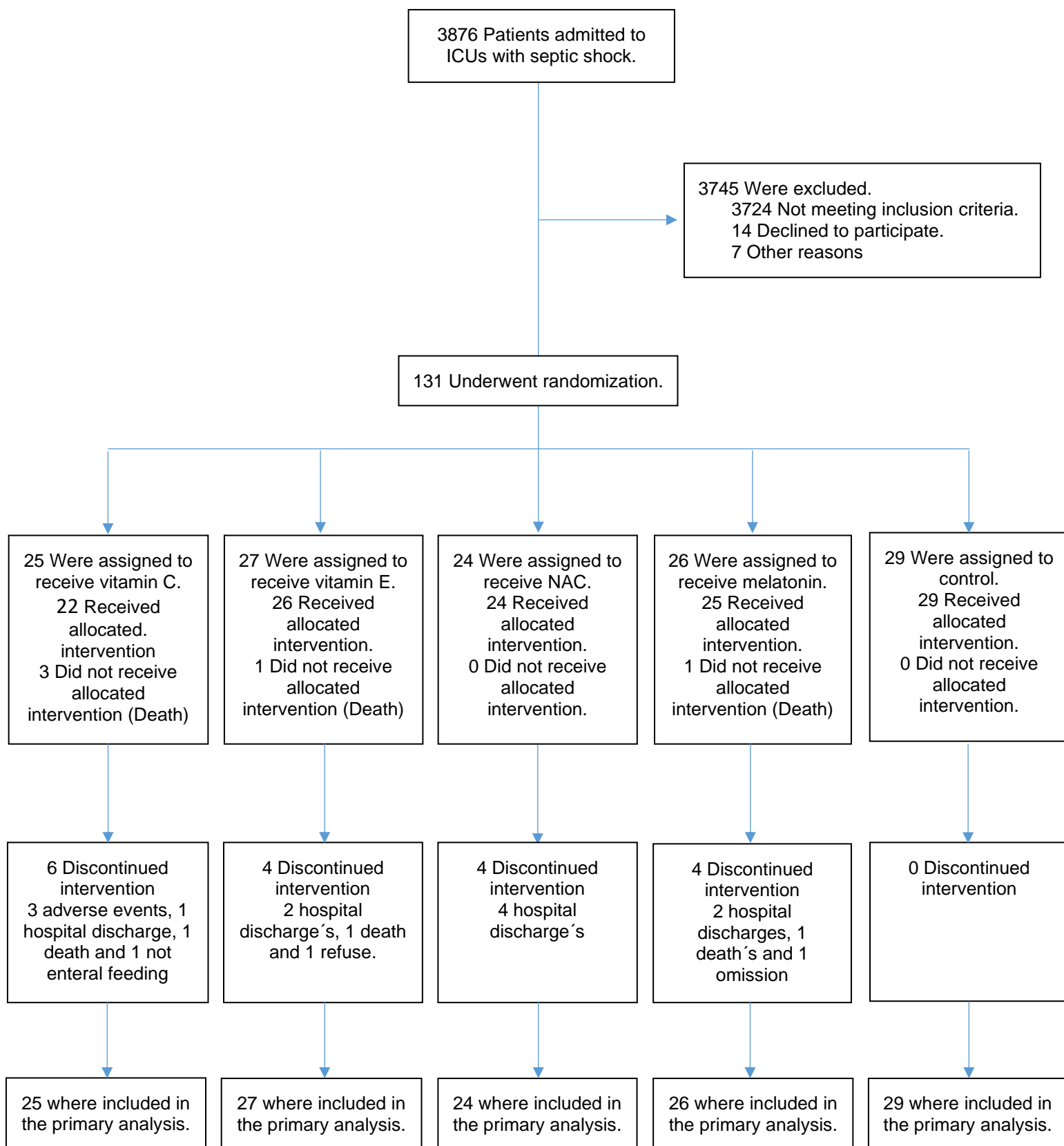

Supplement: Supplementary file 1 [file ijms-24-16610-s001.zip › Supplement S3.pdf]
